# Wave based damage detection in solid structures using spatially asymmetric encoder–decoder network

**DOI:** 10.1038/s41598-021-00326-2

**Published:** 2021-10-25

**Authors:** Frank Wuttke, Hao Lyu, Amir S. Sattari, Zarghaam H. Rizvi

**Affiliations:** 1 Geomechanics and Geotechnics Group, Kiel University, 24118 Kiel, Germany; 2 Competence Centre for Geo-Energy, Kiel University, 24118 Kiel, Germany

**Keywords:** Civil engineering, Computational methods, Scientific data, Computer science

## Abstract

The identification of structural damages takes a more and more important role within the modern economy, where often the monitoring of an infrastructure is the last approach to keep it under public use. Conventional monitoring methods require specialized engineers and are mainly time-consuming. This research paper considers the ability of neural networks to recognize the initial or alteration of structural properties based on the training processes. The presented model, a spatially asymmetric encoder–decoder network, is based on 1D-Convolutional Neural Networks (CNN) for wave field pattern recognition, or more specifically the wave field change recognition. The proposed model is used to identify the change within propagating wave fields after a crack initiation within the structure. The paper describes the implemented method and the required training procedure to get a successful crack detection accuracy, where the training data are based on the dynamic lattice model. Although the training of the model is still time-consuming, the proposed new method has an enormous potential to become a new crack detection or structural health monitoring approach within the conventional monitoring methods.

## Introduction

For the permanent use of existing structures in urban areas, as well as for lifelines and for safety structures such as dams, the successful monitoring of structures is of the highest priority. Usually, conventional methods of structural dynamics are used to analyse the state of structures and to find existing or propagating damages, while wave-based method are less used in ordinary structural dynamics. These methods are more usual in the field of non-destructive testing (NDT)^1,2^ under use of very high excitation frequencies and shorter wave lengths. Beside the type of analysis method—structural dynamics with long wave length or ultra-sound methods in NDT with extreme short-wave length—the matter of the structural analysis is based on the active analysis of excited vibrations or wave fields until the structural damage is detected. The vibration responses that reflects the behavioral discrepancies of a damaged structure can be analyzed in the time, frequency or modal domains. The vibration responses can be measured by various sensors, for example, accelerometers, velocity transducer, displacement sensors, strain gauges, etc., and the measured time domain data can be converted into frequency or modal domain data by transforming techniques. In the past years, a broad range of techniques, algorithms, and systems have been developed for vibration-based damage identification. A number of publications providing comprehensive state-of-the-art reviews on this topic are available^3–5^.

Deep learning methods, especially deep convolutional networks (CNNs or ConvNets), have achieved a great success in various many fields, especially computer vision^6^. CNNs are particularly suitable to learn from array-like data, such as audio, image, and video. These data are uniformly sampled from signals in spatial and/or temporal domains. In many applications, CNNs have been used as the backbone for pattern extraction, such as recognising objects from images^7^, understanding text^8^, and synthesising audio^9^. The huge leap in computer vision leads to many vision-based cracks detection methods. These models are particularly useful in identifying crack existence, types, level of damages, and semantic damage segmentation. The semantic damage segmentation task is to distinguish damaged and non-damaged parts of a surface at a certain spatial resolution. Most of the works employ a symmetric encoder–decoder structure that requires the same spatial dimension of its input and output^10,11^. With the rapid improvements in computational power and advancements in sensor technology, vibration-based crack detection becomes another field where deep learning can play a role^5^. The artificial neural networks (ANN) have long been used to learn the changes of structural response patterns of damaged structures in opposition to non-damaged structures^12^. Compared to the hand-engineered-feature-based methods, the deep-learning-based method uses deep neural networks as a feature extractor to learn representations from wave fields^13^. The structural vibration responses measured from a single sensor is a time-series signal that represented as a 1D-temporal sequence, and can be transformed to a spatially 2D-representation in frequency domain. In order to apply 2-D CNN, the sensory measurements are either rearranged regarding their geometric location, or transformed into frequency domain. Khan et al. transformed displacement and electric potential data into two-dimensional spectral frame representation and then applied a 2D CNN to distinguish between the undamaged and various damaged states^14^. Sun et al. collected response signals of Lamb wave as training data to build a 2D CNN model^15^. The training data needs to be de-noised by a method of the wavelet transformation and transformed into a two-dimensional spectral frame as the input of the 2D CNN model. The resulted model is able to distinguish the characteristics of the signals between various damage locations at a very high recognition rate. The 2D CNN is also used to predict the bounding box of a crack from raw strain field^16^. By adding noise and changing loading patterns to augment the data, the model could achieve some robustness. Sajedi and Liang proposed a fully convolutional encoder–decoder neural network to perform semantic damage segmentation^17^. The task of semantic damage segmentation is to monitor damage in a grid environment of a large number of nodes. The model requires the same spatial extent of the sensory grid and the structural surface. The consideration of “null” node enhances the model's robustness and maintains the input shape of the model.

1D CNNs and RNNs are two popular structures to recognize patterns from 1D signals. Abdeljaber et al. trained multiple 1D CNNs to detect whether damage exists at specific locations (joints)^18^. Their model uses the acceleration signal at each joint as input and requires extra workload to segment the signal into frames. Considering damage detection as a binary prediction problem, i.e., predicting whether a crack exists from input data, 1D CNN, RNN, and LSTM models all can achieve high accuracy^19^. In one following paper, the authors developed a two-stage damage detection method^20^. The method determines whether a sample is damaged or not at the first stage and then predict the location and length of the damage with another regressor network. However, the regressor network deals only with the damage that is orthogonal to the sample's surface. By coupling the vibration response of undamaged and fully damaged structures, the authors can train for each possible damage location in a 1D-CNN model with much less data^21^. Lin and his colleagues investigated damage detection using low-level waveform signals for a simply supported beam^22^. They segmented the simulated time-series signals and added noise to augment the dataset. The 6 stacked 1D-ConvLayers were used as feature extractor. The model achieves a high accuracy for single and multiple damage, and is robust to noise. Rai and Mitra proposed a multi-headed 1D-CNN architecture for damage detection based on raw discrete time-domain Lamb wave signals recorded from a thin metallic plate^23^. Both simulated data and experimentally generated data are used to train and evaluate the model. The model performs well to detect notch-like damage on both datasets. Besides this method, there is also an attempt to use unsupervised methods to identify undefined damages based on the features that are extracted by CNNs^24^.

In general, existing vibration-based methods have proved the importance of 2D-CNN and 1D-CNN in building deep learning models for crack detection. However, most studies focus on the damage identification, i.e., determination of damage occurrence; a few efforts are made to identify damage’s geometric locations, but at restricted scenarios. The spatial asymmetric encoder–decoder network (SpAsE-Net) presented in this work contributes to solving semantic crack segmentation task from a vibration-based perspective. Comparing to the previous methods, it requires a spatially sparse sensor grid and is able to identify the damages for the whole sample surface.

Training the deep learning models requires a large amount of data. For wave-based crack detection models, these necessary data can be generated from numeric simulations such as Finite Element Method (FEM)s, or from the sensory measurements of a lab/field configuration^5^. The numerical treatments for the paper are done by use of a meso-scale method as it is better suited to capture the effects as initial cracking and crack propagation depending on the material parameter and the initial- \& boundary conditions without pre-definition of damaged pixels, and it is also applicable for 2D and 3D problems. The Lattice Element Method is a class of discrete models in which the structural solid is represented as a 3D assembly of one-dimensional elements^25–27^. This idea allows one to provide robust models for propagation of discontinuities, multiple crack interactions, or cracks coalescence even under dynamic loads and wave fields. Different computational procedures for lattice element methods for representing linear elastic continuum have been developed. Beside different mechanical, hydro-mechanical and multi-physical developments, the extension and basics for a new dynamic Lattice Element Method was proposed as well^28,29^. This development will be used in the given paper for the health monitoring of structures.

To perform the damage detection as a numerical software, pattern indicators and specific designed deep networks (DNN) are needed. The numerical simulation is realized under use of Dynamic Lattice-Element Method, where the advantage of the discontinuum method in opposition to continuum methods related to the damage detection will be discussed in the methodology section. The implemented artificial neural networks are also described in this section. Based on the considered numerical and DNN models, a case study of a 2D plane is performed to show the developments and results of the new approach.

## Methods

### Dynamic lattice approach

The assembly of the heterogeneous and homogeneous material will be generated by specific meshing algorithms in LEM. The Lattice Element Models with the lattice nodes can be considered as the centers of the unit cells, which are connected by beams that can carry normal force, shear force and bending moment. Because the strain energy stored in each element can be exceeded by a given threshold, the element is either removed for cracking or assigned a lower stiffness value. The method is based on minimizing the stored energy of the system. The size of the localized fracture process zone around the static or propagating crack plays a key role in failure mechanism, which is observed in various models of linear elastic fracture mechanics and multi-scale theories or homogenization techniques. Normally this propagating crack process needs a regularization, however, an efficient way of dealing with this kind of numerical problem is by introducing the embedded strong discontinuity into lattice elements, resulting in mesh-independent computations of failure response. The generation of the lattice elements are done by Voronoi cells and Delaunay itself^27,30^. With the performance of this procedure an easy algebraic equation is generated for the static case. To develop the dynamic LEM for simulation of a propagating wave field, a more complex extension of the LEM is needed. The following solution of the dynamic LEM is solved as a transient solution in the time domain.

### Equation of motion

To solve the dynamic LEM, the static LEM needs an extension of the equation of motion. The general equation of motion without the damping term is defined by

1 $${\varvec{M}}\ddot{u} + {\varvec{K}}u = F(t)$$

where ${\varvec{M}}$ and ${\varvec{K}}$ are the mass and the stiffness matrices terms and $F\left(t\right)$ is the applied time-dependent force. Both matrices, the mass and stiffness matrix, have to be defined in terms of the LEM definition.

### Mass matrix generation

The mass matrix or the consistent mass matrix (CMM) is generated either by lumping the mass at the nodes or by following the variation mass lumping (VMM) scheme. The VMM scheme is also implemented in the finite element method for dynamic simulations.

The element mass ${M}^{e}$ is computed using the following equation

2 $$M^{e} = \int \rho \left[ {N_{v}^{e} } \right]^{T} N_{v} d\Omega$$

If the shape functions are identical, that is, ${N}_{v}^{e}={N}^{e}$, the mass matrix is called the consistent mass matrix (CMM) or ${M}_{\mathrm{c}}^{\mathrm{e}}$.

3 $$M_{c}^{e} = \int\limits_{0}^{1} {\uprho } {\text{A}}\left. {\left[ {{\text{N}}^{{\text{e}}} } \right.} \right]^{T} N^{e} dx = \frac{1}{4}\rho lA\int\limits_{0}^{1} {\left[ {\begin{array}{*{20}c} {1 - \epsilon } \\ {1 + \epsilon } \\ \end{array} } \right]} \left[ {1 - \epsilon \quad 1 + \epsilon } \right]d\varepsilon$$

where, $\uprho$ is the density assigned to the Voronoi cells, and $\mathrm{A}$ and $l$ are the area and the length of the lattice elements. The elemental mass matrix is symmetric, physically symmetric, and complies with the condition of conservation and positively. To obtain the global mass matrix, a congruent transformation is applied. In contrast to the stiffness matrix, translational masses never vanish. All the translational masses are retained in the local mass matrix. The global transformation is achieved through the following equation.

4 $$\bar{M}_{c}^{e} = \left. {\left[ {{\text{T}}^{{\text{e}}} } \right.} \right]^{T} \left[ {M_{c}^{e} } \right]\left[ {T^{e} } \right]$$

5 $$\begin{array}{*{20}c} {{\text{M}}_{{\text{c}}}^{{\text{e}}} = \frac{1}{2}{\text{m}}^{{\text{e}}} \left[ {\begin{array}{*{20}c} 1 & 0 & 0 & 0 \\ 0 & 1 & 0 & 0 \\ 0 & 0 & 1 & 0 \\ 0 & 0 & 0 & 1 \\ \end{array} } \right]} \\ \end{array}$$

### Element stiffness matrix

The force displacement component of a truss element is given by the spring relation

6 $$\left\{ F \right\} = \left[ K \right]\left\{ U \right\}$$

The vectors $\left\{F\right\}$ and $\left\{U\right\}$ are the member joint force and member joint displacement. The member stiffness matrix or the local stiffness matrix is $\left[K\right]$. For a truss element it is given by

7 $$\begin{array}{*{20}c} {\left[ K \right] = \frac{{EA}}{L}\left[ {\begin{array}{*{20}c} 1 & 0 & { - 1} & 0 \\ 0 & 0 & 0 & 0 \\ { - 1} & 0 & 1 & 0 \\ 0 & 0 & 0 & 0 \\ \end{array} } \right]} \\ \end{array}$$

After applying the congruent transformation, the member stiffness matrix in global coordinates are given as

8 $$\begin{array}{*{20}c} {\left[ {K^{e} } \right] = \left[ {T^{e} } \right]^{T} \left[ K \right]\left[ {T^{e} } \right]} \\ \end{array}$$

9 $$\begin{array}{*{20}c} {K^{e} = \frac{{E^{e} A^{e} }}{{L^{e} }}\left[ {\begin{array}{*{20}c} {l^{e} } & {lm} & { - l^{2} } & { - lm} \\ {lm} & { - m^{2} } & { - lm} & { - m^{2} } \\ { - l^{2} } & { - lm} & {l^{2} } & {lm} \\ { - lm} & { - m^{2} } & {lm} & {m^{2} } \\ \end{array} } \right]} \\ \end{array}$$

where $l=cos{\upphi }^{e}$, $m=sin{\phi }^{e}$, and with ${\phi }^{e}$ as the orientation angle.

### Time domain solution of equation of motion

The equation of motion for the linear system of equations is solved with the Newmark beta method due to its unconditional stability. The displacement and the velocity terms for the next time step are calculated as follows:

10 $$u_{t} = u_{{t - \Delta t}} + \Delta t\dot{u}_{{t - \Delta t}} + \left( {\frac{1}{2} - \upbeta } \right)\Delta t^{2} \ddot{u}_{{t - \Delta t}} + \beta \Delta t^{2} \ddot{u}_{t}$$

11 $$\begin{array}{*{20}c} {\dot{u}_{t} = \dot{u}_{{t - \Delta t}} + \left( {1 - \upgamma } \right)\Delta t\dot{u}_{{t - \Delta t}} + \gamma \Delta t\ddot{u}_{t} } \\ \end{array}$$

We follow the average acceleration approach with $\upbeta =\frac{1}{4}$ and $\upgamma =\frac{1}{2}$ .

The Newmark beta method solves the algebraic form of the equation of motion (EOM) of undamped forced vibration at the end time interval $t+\Delta t$

12 $$F_{{t + \Delta t}} = {\varvec{M}}\ddot{u}_{{t + \Delta t}} + {\varvec{K}}u_{{t + \Delta t}}$$

The stiffness and the mass matrices are computed in the following fashion to reduce in the form of Eq. 6.

13 $${\hat{\varvec{K}} = {\varvec{K}} + a_{0} {\varvec{M}}}$$

where $$\widehat{{\varvec{K}}}$$ $ is the effective stiffness matrix and $${\mathrm{a}}_{0}=\frac{6}{\mathrm{\gamma \Delta }{\mathrm{t}}^{2}}$$

Similarly, the effective load vector at time $t+\Delta t$ is calculated as

14 $$\hat{F}_{{t + \Delta t}} = F_{{t + \Delta t}} + M\left( {a_{0} u_{t} + a_{2} \dot{u}_{t} + a_{3} \ddot{u}_{t} } \right)$$

Here, $${\mathrm{a}}_{2}=\frac{1}{\mathrm{\gamma \Delta t}}$$ and $${\mathrm{a}}_{3}=\frac{1}{2\upgamma }$$.

The above simplification leads to the algebraic form.

15 $$\left\{ {\hat{F}_{{t + \Delta t}} } \right\} = \left[ {\hat{K}} \right]\left\{ {U_{{t + \Delta t}} } \right\}$$

From the above equation, displacement of each node is calculated for every time step. The natural frequency of the system is calculated as given below.

16 $${\upomega ^{2} \left[ M \right]\Phi = \left[ K \right]}$$

17 $${\upomega ^{2} = eig\left( {\left[ M \right]^{{ - 1}} \left[ K \right]} \right)}$$

The detailed description of the theory and implementation of the dynamic Lattice-Element method with validation and verification of the method by analytical and numerical benchmarks is given in^28,29^.

### Wave field identification by convolutional neural networks

#### The wave field dataset

##### Numerical simulation of wave field

The basic idea of developing deep learning models for damage detection in the given case of propagating wave fields is the identification of wave field patterns respective of the change in wave field patterns during the damage evolution. The damage evolution process covers the initial static case on the given plate. After a change of surrounding, static stress condition damages on different positions in the plate area can be created depending on the stress condition and the material parameter. Before and after a damage scenario, a small strain wave field is excited to propagate through the plate. Because of the damage/crack, within the plate the pattern of the propagating wave field will be modified. The interaction of the wave field within the crack is essential for identifying the correct wave field. Under the assumption of an open crack, neglecting shear slipping and crack growth under dynamic loads, the crack will produce a mode conversion and a scattering of the propagating wave field^28,29^. It becomes obvious that the transient solution provides that phenomena.

In this paper, the whole content of the wave field, the initial wave front, the coherent part and the diffusive part will be used for the damage detection in the time domain. There is no selection respective analysis of harmonic wave modes in the part coherent wave field^31^ or application of the interferometric method at the code in the diffusive part^32^ of the wave field yet. The sequential measures of time-dependent displacement amplitudes are used as the input data for the proposed damage detection network. The instantaneous load added at a chosen excitation point causes high displacement amplitudes at the wave field and decreases rapidly after several time steps to the wave coda at a smaller strain level. The observable surface wave front, as well as at interference of back scattered and reflected wave field is the result of the wave propagation and reflection in the plate. The described phenomenon is clearly visible in an example in the corresponding part in “Result” section.

##### Generation of the damage detection dataset

**Figure 1:**
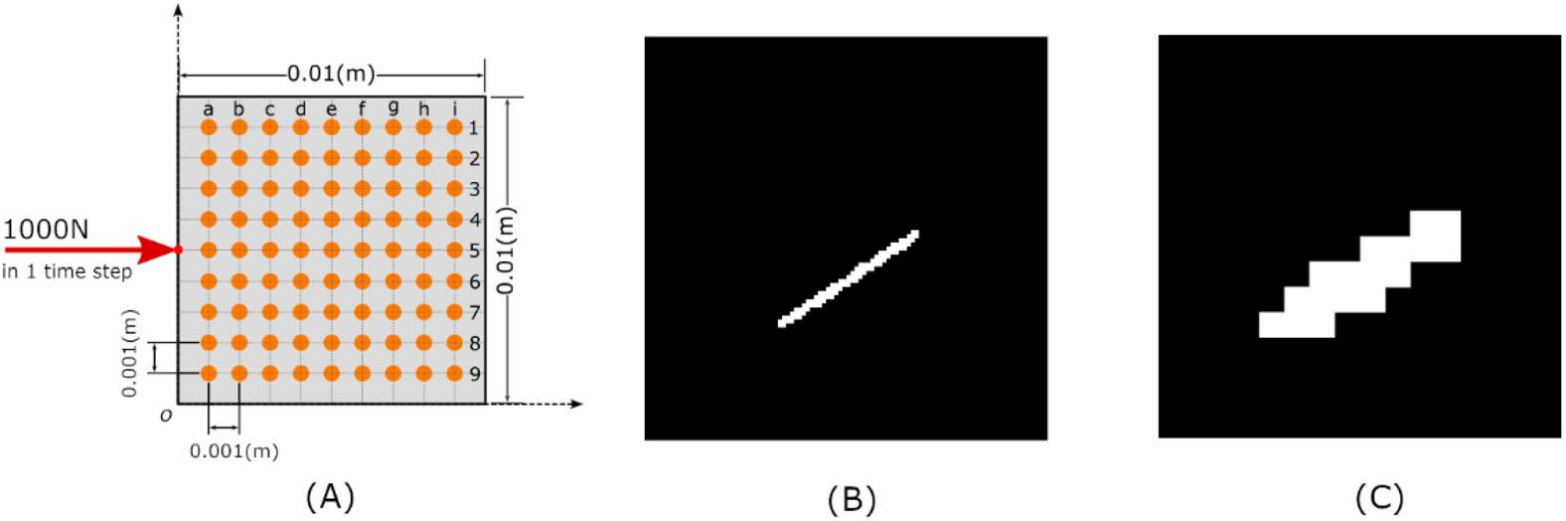
Configurations for the dataset: (**A**) The receiver arrangement on the surface of a plate; (**B**) a 100 × 100 binary labelling image; (**C**) the corresponding 16 × 16 binary labeling image.

To generate the training dataset, the following variables are kept constant, including the excitation and receiver points, the size of sample plates, the impulse load and time span, and the Young's modulus of a plate. The detailed variables for running simulations are described in the corresponding part in “Result”. The plate, receivers and excitation points are shown in Fig. 1A. The resulted displacement wave field consists of time histories in the X- and Y-direction at 81 receiver positions with 2000 time-steps, i.e., an array of shape $2000\times \left(9\times 9\right)\times 2$.

To validate the damage detection method with randomly generated cracks, the crack itself is described by 3 parameters with randomly chosen values, i.e., crack length $l\in \left(0,\frac{1}{2}\mathrm{min}\left({e}_{x},{e}_{y}\right)\right]$, orientation $\mathrm{\alpha }\in [0, 360]$, and start position $\left(\mathrm{x},\mathrm{y}\right)$, where $x\in \left[{s}_{x},\hspace{0.17em}{e}_{x}-{s}_{x}\right]$, $y\in \left[{s}_{y},\hspace{0.17em}{e}_{y}-{s}_{y}\right]$, ${e}_{x}$ and ${e}_{y}$ are the length of the sample edges along the x-axis and y-axis, ${s}_{x}$ and ${s}_{y}$ are the distance between two receivers in the X-axis and Y-axis (see Fig. 1 A). If one randomly generated crack stretches out of the sample plate, the excess part is discarded. The plate particles that correspond to the crack are marked as removed for the Lattice-Element model calculation.

Binary labelling images are generated to facilitate supervised training of the model. The binary image covers the plate's surface and indicates whether crack exists within a pixel's area (pixel value equals to 1, otherwise 0). The labelling image is first obtained of an $100\times 100$ resolution, where each pixel covers an area similar to the size of the lattice element in a sample. When the model is adjusted to refine or enlarge predictions, the resolution of the label image can be changed accordingly. Figure 1B,C gives two label images of different resolutions for the same plate. The image of $16\times 16$ resolution is resized and binarized from the image of $100\times 100$ pixels. We use the low-resolution images as supervision signal to restrict the problem scale while maintain the model's applicability.

#### The SpAsE-Net with 1D-CNN Wave pattern extractor

##### The network structure

The proposed model makes binary classification for each pixel w.r.t. the labelling image to decide where damage exists, which is comparable to binary image segmentation task, i.e., distinguish between foreground pixels and background pixels^33^. Its major difference from an image segmentation model is to infer the spatial distribution of damage from spatially sparse sampled temporal data instead of making spatial-to-spatial transformation.

**Figure 2:**
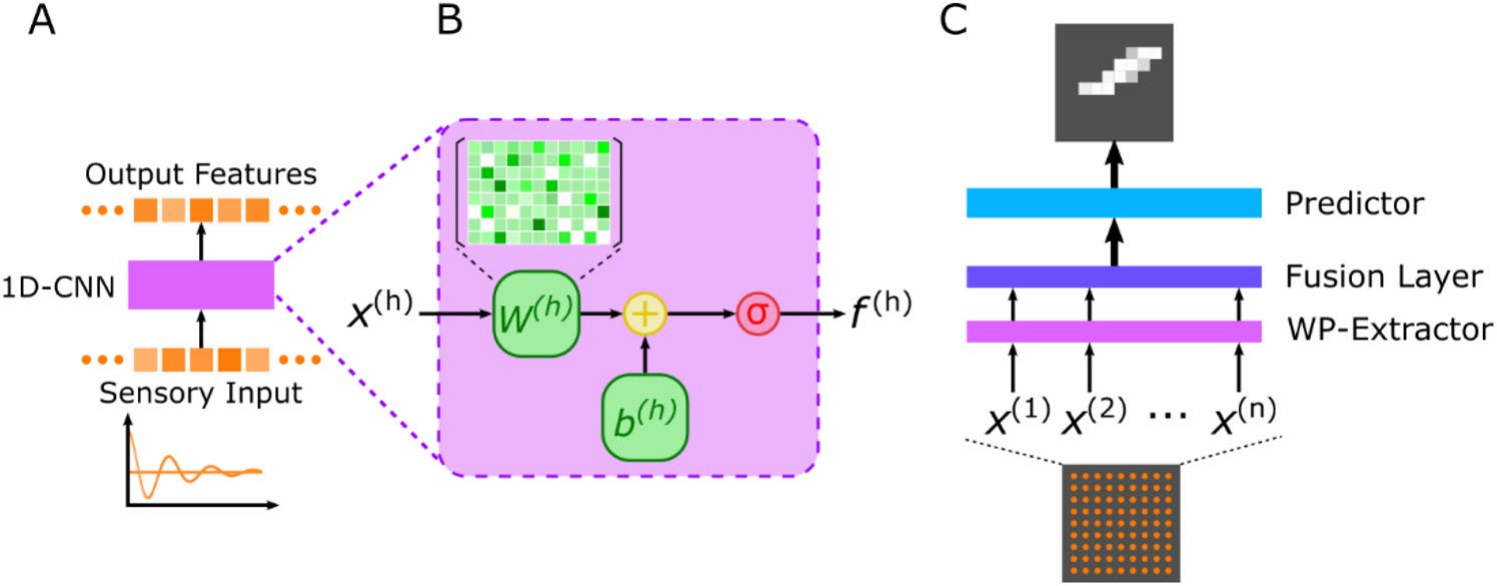
Conceptual explanation of the proposed crack detection model with 1D-CNN detector. (**A**) Diagram of a 1D-CNN that transforms a discretized wave input and produces a discrete feature sequence. (**B**) Internal STRUCTURE of a 1D-CNN layer, consisting of a trainable weight ${W}^{\left(h\right)}$, a bias ${b}^{\left(h\right)}$. Activation function is represented by $\sigma$. C. Diagram of the complete structure of the proposed model.

The model has three components: a set of 1D-CNN layers acting as a wave pattern (WP) extractor to learn features from wave fields histories; two fully convolutional layers to fuse WPs from the temporal dimension and the receiver's dimension (spatial); and a predictor module taking the fused features as input and making predictions of crack existence (Fig. 2). The input wave field histories—typical time series—are suitable for 1D-CNN layers to handle.

##### Implementation details

CNN is particularly useful to analyse natural signals in spatial and temporal domain^34^. It is a feed-forward network that consists of trainable multistage feature extractors. The training process is an optimization procedure, where the gradient of an objective function with respect to the weights of the feature extractors is calculated and back-propagated^35^. In the implementation, CNN differentiates itself from other ANNs by using the local connection (one “neuron” connects locally with only a restricted number of “neurons” in its previous layers,), weight sharing, and pooling strategies^6^. A typical CNN architecture is composed of a set of convolutional layers (ConvLayer), and then fully connected layers or some more ConvLayers. ConvLayer detects local features from the previous layer by transforming the input signal with a set of convolutional kernels (filters). It produces different kinds of feature maps with respect to its filters, then an activation operation is applied to the feature maps. The non-linear activation functions “squeeze” the values of a feature maps into a certain range, for example $\left[0, 1\right]$ for Sigmoid function, $\left[-0.5, 0.5\right]$ for Tanh and $\left[0,+\infty \right)$ for Rectified Linear Unit (ReLU). Pooling layer is used for down-sampling the feature maps by taking local average or local maximum values. The pooling layer merges semantically similar features into a higher level^6^. However, pooling layers can be intentionally replaced by setting a larger stride in the convolution layer^36^.

**Figure 3:**
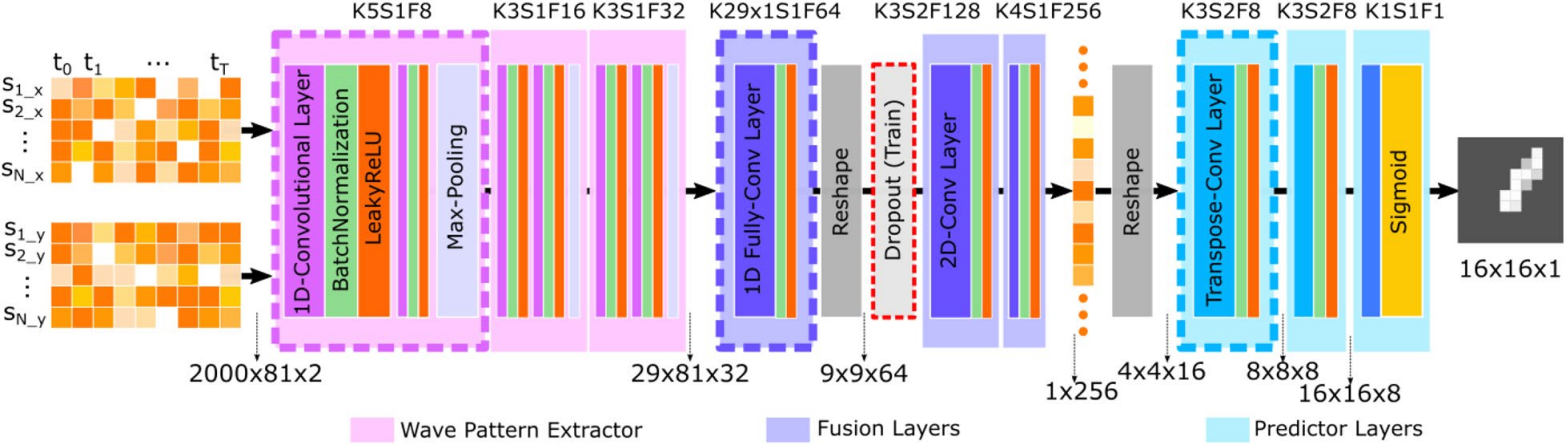
Detailed design of the damage detection model. The shape of input data and layer output is placed at the bottom; layer configurations are placed on top for each layer.

The detailed implementation of the model is shown in Fig. 3 The WP-Extractor consists of three 1D-ConvLayer blocks. Each block has two 1D-ConvLayers followed by a BatchNormalization layer^37^ and an activation layer—using the LeakyReLU function^38^. The BatchNormalization layer standardizes the output of the ConvLayer by re-scale according to the samples in a batch. It prevents dramatic change of the spread and distribution of inputs during training, and has the effect of stabilizing and speeding-up the training process. LeakyReLU is a variation of ReLU. The ReLU function results in zero when the input is less than zero and keeps the input unchanged when the input is above or equal to zero. The LeakyReLU function “squeezes” the value when the input is less than zero and thus allows a small, non-zero gradient when the unit is not active. The two ConvLayers in the same block share identical kernel size and filter numbers. The output of each block is passed through a MaxPooling layer to reduce the data size in time dimension.

The first fusion layer reduces the temporal dimension to 1 with a 1D fully convolutional layer. Then the transformed feature maps are reshaped according to the spatial position of the receivers (see Fig. 1A). Two 2D ConvLayers receive the transformed feature map and combine the information from all receivers. The output of the fusion block is a 256-dimension vector. To reduce overfitting and further improve model performance, a dropout layer is added after the first fusion layer^39^. The Dropout layer drops the extracted features from randomly selected receiver locations during training. It forces the model to learn the wave field dynamics from less receiver locations. The influence of using dropout can be found in the Supplementary.

The core module of Predictor is composed of two Transpose-Convolutional layers (TransConvLayer, sometimes misinterpreted as deconvolution). TransConvLayer is a widely used upsampling technique for image segmentation and image generation^40,41^. The TransConvLayers up-sample the fused information to desired spatial resolution. The final layer, a 2D ConvLayer transforms the channel-wise information to a single value and uses sigmoid function make predictions.

The layer configuration and wave pattern shapes are also shown in Fig. 3, K refers to the kernel size, S is the steps, F indicates the number of filters. The step of Max-Pooling is set to 4. The 1D convolution can be implemented using 2D convolution by fixing the kernel size of the receiver dimension to 1.

##### The loss function

Training the proposed model is an optimization procedure, and relies on the objective function/loss function. In this work, the proposed model makes multiple binary predictions for every pixel per each sample. For single binary classification problems, cross entropy (CE) loss (see Eq. 18, where label $\mathrm{y}=\{0, 1\}$, $p$ is the predicted probability.) may be the most commonly used loss function. However, the “has crack” pixels consist only a very small portion of total pixels. CE loss can introduce bias towards “no crack” predictions, i.e., simply predicting all pixels as “no crack” already result a rather low loss value. To tackle such extreme class imbalance, we select Focal Loss (FL) as our loss function. FL was originally proposed to address the extreme class imbalance in object detection^42^. FL is a variation of CE loss by adding a penalty term to reduce the loss value of already correctly predicted training cases. The penalty term ${\left(1-p\right)}^{\upgamma }\left(\upgamma \ge 0\right)$ re-weights between difficult and easy examples. During training, if a sample is already predicted correctly with a high probability, it is called an “easy” case. The penalty term reduces its loss and thus focus on “hard” cases, where correct predictions are made with a much lower probability.

18 $${L_{{{\text{CE}}}} = - \left( {y{\text{log}}\left( p \right) + \left( {1 - y} \right){\text{log}}\left( {1 - p} \right)} \right)}$$

To adjust the loss values of the two binary classes, a weighting factor $\mathrm{\alpha }\in \left[\mathrm{0,1}\right]$ can be added. Similar to defining ${p}_{t}$, ${\mathrm{\alpha }}_{t}$ can be defined as $\mathrm{\alpha }$ for class 1, and $1-\mathrm{\alpha }$ for class 0. For the crack detection case, let ${y}_{\left\{k,i,j\right\}}$ denote the binary label image of sample $k$ and ${p}_{t\left\{k,i,j\right\}}$ the prediction for the correspoinding pixel $\left({i}^{th},{j}^{th}\right)$ of the label image. The loss on cases $k$ is calculated by:

19 $$\begin{array}{*{20}l} {L_{{{\text{FL}},k}} = \frac{1}{{U*V}}\sum\limits_{{i = 1}}^{U} \sum\limits_{{j = 1}}^{V} - \alpha _{t} \left( {1 - p_{{t\{ k,i,j\} }} } \right)^{\gamma } log\left( {p_{{t\{ k,i,j\} }} } \right),\left\{ {\begin{array}{*{20}l} {p_{t} = p,\alpha _{t} = \alpha } & {if\;y = 1} \\ {p_{t} = 1 - p,\alpha _{t} = 1 - \alpha } & {othterwise} \\ \end{array} } \right.} \\ \end{array}$$

where $U$, $V$ are the number of columns and rows of the label image.

Two hyper-parameters are introduced by Focal loss, $\mathrm{\alpha }$ and $\upgamma$. When $\upgamma =0$, FL is equivalent to weighted CE. When $\upgamma$ increases, the modulating factor uses a lower standard to define easy examples and has greater power to down-weights well-classified examples. In practice, the optimal $\mathrm{\alpha }$ and $\upgamma$ are found out by empirical studies.

#### Training process

##### Data pre-processing

In this simulation, the recorded data at the edge receivers are discarded to avoid any possible effects caused by the extremely large values. Thus, in total the records at 81 receivers are used for both training and testing. Then the wave displacements are normalized between -1 and 1 according to each sample's maximum and minimum value. The resulting input data for the CNN model is $2000\times 81 \times 2$ matrix for each case.

##### Training configurations

Gradient based methods are the most common ways to optimize a neural network. In recent years, a variety of algorithms have been developed to achieve efficient, robust, and stable training of DNNs. Momentum and Nesterov gain faster convergence and more stability by adding a momentum term to SGD (stochastic gradient descent, which estimates the gradient from a randomly selected subset of data). The family of adaptive learning rate methods, including AdaGrad, AdaDelta, RMSprop, and Adam, applies low learning rates for parameters associated with frequently occurring features and higher learning rates for parameters associated with less frequent features. These methods eliminate the need to manually tune the learning rate. Ruder provides a comprehensive overview of these optimizer algorithms^43^. In this study, Adam is chosen as the optimizer. The Adam optimizer computes the exponentially decaying average of past squared gradients like AdaDelta and RMSprop, and also computes exponentially decaying average of past gradients similar to momentum. Adam optimizer is proved to work well in practice through empirical studies^44^. In many cases, as well as this study, a default learning rate of 0.01 can work well. A discussion on learning rate can be found in Supplementary. The training epochs were set to 150 for all experiments to ensure sufficient training steps for the models to converge. The best model with respect to the evaluation metric is saved for evaluation.

The optimal hyper-parameters, $\mathrm{\alpha }$ and $\upgamma$ are determined by an ablation study. The detailed result for alpha and gamma is listed in Results section.

The proposed model is implemented in Python with Tensorflow 2.1 Keras. The ablation experiment is performed on a workstation of Windows 10 with Nvidia GPU.

##### Evaluation metrics

We use the value of intersection over union (IoU), the Dice similarity coefficient (DSC), and an IoU-based accuracy for model evaluation. Although the loss values indicate the quality of the prediction on the patch basis, predicting cracks can be more focused. The predicted crack pixels and true crack pixels consists two sets. The intersection of the two sets is all successfully found damaged pixels. The union of the two sets is the sum of predicted damaged pixels and not detected damaged pixels. High IoU value indicate that the “has crack” pixels covers more damaged pixels, and as few “no crack” pixels.

The correctly predicted damaged/not-damaged pixel, is marked as TP/TN; the wrong predictions on damage existence, is either FP or FN as shown in Table 1. Based on this, we can calculate precision ($TP/\left(TP+FP\right)$) and recall ($TP/\left(TP+FN\right)$) as well as IoU and DSC,

**Table 1:** Prediction typology in a binary-classification-based damage detection.

	True Prediction (T)	False Prediction (F)
Has Damage (P)	TP	FP
No Damage (N)	TN	FN

The IoU metric calculates the ratio between the intersection and the union of ground truth damage area and predicted damage area using Eq. 20. Similarly, the DSC metric is calculated using Eq. 21. Both DSC and IoU metric range between 0 and 1. If there is no overlap between predicted damaged pixels and true damaged pixels, both metrics are equal to 0. When predicted crack pixels covers more true damaged pixels, the intersection area becomes larger and the union area becomes smaller, resulting in a value closer to 1. When predicted crack pixels covers exactly the true damaged pixels, DSC and IoU metrics reach their upper limit of 1. When the sample has no damage and the model makes correct predictions, i.e., both the intersection and the union are 0, both DSC and IoU metrics are assigned the value of 1.

20 $${M_{{IoU}} = \frac{{area\left( {TP} \right)}}{{area\left( {TP} \right) + area\left( {FP} \right) + area\left( {FN} \right)}}}$$

21 $${M_{{DSC}} = \frac{{2area\left( {TP} \right)}}{{2area\left( {TP} \right) + area\left( {FP} \right) + area\left( {FN} \right)}}}$$

Since we have an underlying condition that states each sample has maximum “one crack” inside. We can define the “accuracy” using IoU values. For a single prediction of a sample, we consider it as “correct” if its IoU is greater than a given threshold. Given the threshold, the accuracy on the whole dataset is calculated as the ratio of the number of samples whose IoU value is greater than the threshold, to the total number of evaluated datasets.

## Results

### Simulated displacement wave field using dynamic lattice element method

**Figure 4:**
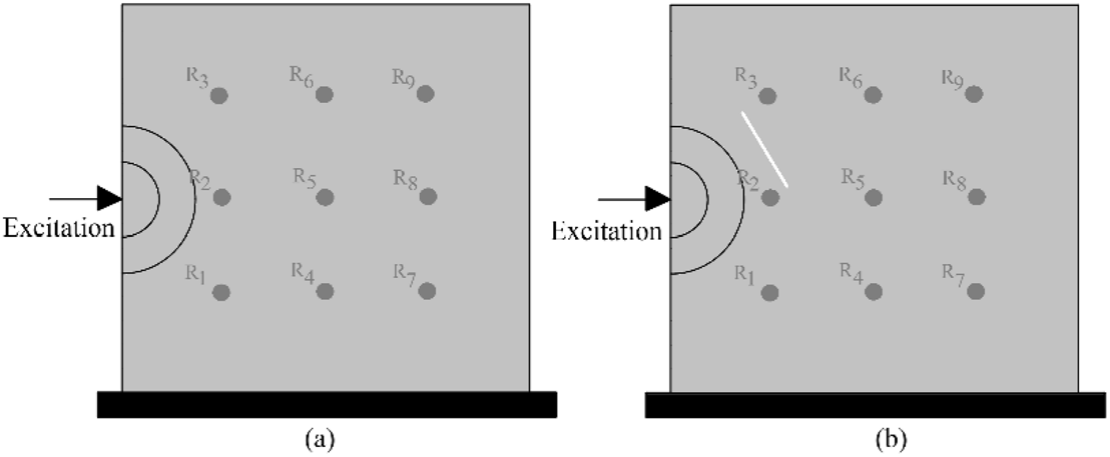
The boundary conditions and assigned reference points: (**a**) horizontal excitation without generated crack; (**b**) horizontal excitation with generated crack.

We developed a dynamic lattice element method to simulate the wave fields in a 2D plate. The considered boundary conditions with excitation points and crack conditions are shown in Fig. 4.

**Figure 5:**
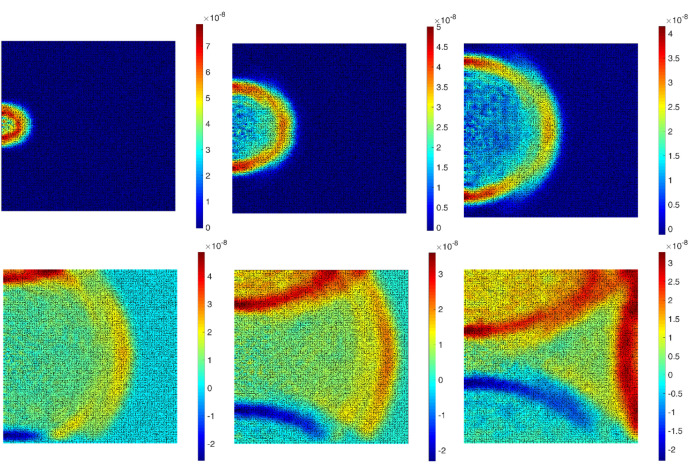
The 6 frames (100 time-steps interval, from left to right) of a displacement (${u}_{x}$) wave propagation inside the defined plate in Fig. 4a.

**Figure 6:**
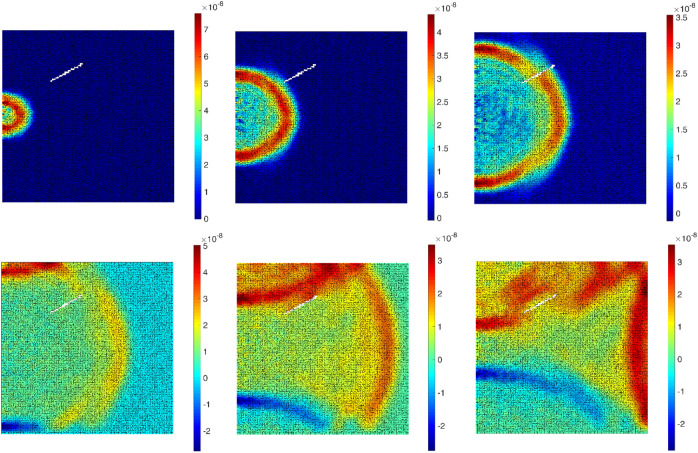
The 6 frames (100 time-steps interval, from left to right) of a displacement (${u}_{x}$) wave propagation inside the defined plate in Fig. 4b.

Figure 5 shows the simulated wave fields in lateral direction for a boundary condition of Fig. 4 a. The simulated wave fields with a generated crack (Fig. 4b) are shown in Fig. 6. For another conditions in Fig. S1—excitation point in upper middle boundary—the wave field is plotted in Fig. S2. The results clearly show wave shadows behind the crack as well as the reflection of the wave field from the defined cracked surface.

**Figure 7:**
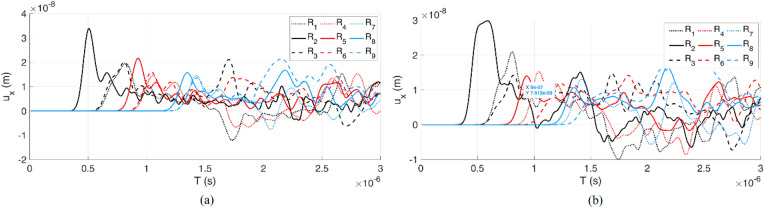
The time histories (${u}_{x}$) of the reference points inside the plate: (**a**) without crack, (**b**) with a generated crack.

The time histories (${u}_{x}$) of the reference points (${R}_{1:9}$) inside the plate (Fig. 4) are shown in Fig. 7. Two boundary conditions are considered: one with discontinuity (crack), and one without. The plate dimension is 10 × 10 cm and the load excitation is at the left middle boundary. The applied rectangular impulse load with a magnitude of 1 $kN$ is kept for 10 time-steps, where $\Delta t = 0.00000001 s$. The Young's modulus of a plate is assigned to 5 $\mathrm{GPa}$.

Figure 7 clearly shows the arrival time of the wave fields at each reference point. The closest reference point (${R}_{2}$) has the maximum amplitude and minimum arrival time. In Fig. 7 a, the arrival time of the wave field to ${R}_{1}\approx {R}_{3}$, ${R}_{4}\approx {R}_{6}$ and ${R}_{7}\approx {R}_{9}$. Due to the generated discontinuity (crack) in Fig. 7 b, the first arrival times of the wave field to ${R}_{3}$, ${R}_{6}$ and ${R}_{9}$ are delayed. Theses reference points are located in the shadow field behind the generated discontinuity. Having a closer look at the ${R}_{2}$, it is obvious that due to the wave reflection from the generated discontinuity, the arrival of the second wave field happens sooner than the first boundary condition, approximately $1.45\times {10}^{-6}s$. The length, location and orientation of the discontinuities affect the wave field in the domain. The simulated wave fields at the reference points are used for training and developing the artificial neural network model.

### Damage detection dataset

In total, we generated 3040 samples for training and 320 samples for testing. There are different types of samples with respect to the randomness of sample generation and crack generation. The plate and crack of *Type-N* samples are both generated randomly. The reference samples without any crack inside are marked as *Type-R*. The samples of different plates with similar cracks (*Type-S*) and the same plates with different cracks (*Type-C*) are also generated. Fig. S4 shows a detailed distribution of these 4 types in test dataset. Among all test samples, we intentionally generated 7 random samples, and each one has its counter case in the training dataset in terms of the same crack (as well as no-crack cases). It is worth emphasizing that these samples are not repeated ones. Because of the randomness in the generation process, the diversity of the interior particles and their wave field patterns is ensured.

#### The SpAsE-Net for damage detection

##### Identified optimal hyper-parameters for the SpAsE-net

**Figure 8:**
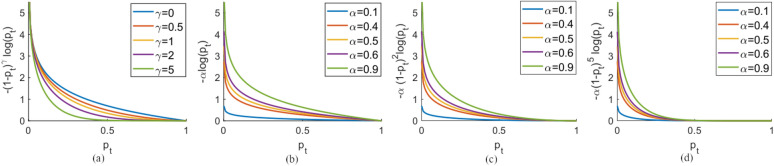
The focal loss values with different $\gamma$ s and $\alpha$ s. (**a**). Focal loss without weight $\alpha$. (**b**). Focal loss with different $\alpha$ value with fixed $\gamma =0$. (**c**). Focal loss with different $\alpha$ value with fixed $\gamma =2$. (**d**). Focal loss with different $\alpha$ value with fixed $\gamma =5$.

Focal Loss (FL) introduces two hyper-parameters $\upgamma$ and $\mathrm{\alpha }$. They are used to adjust the loss value of a prediction during the training, so the training can focus on specific types of training cases. Figure 8 shows the FL value with different $\upgamma$ and $\mathrm{\alpha }$ values. Figure 8 a is a remake of Fig. 1^42^. With use of the penalty term, the loss value is reduced with the probability of making correct predictions increasing. $\upgamma$ controls the decay strength, and larger $\upgamma$ ensures the loss to decrease faster. For example, when $\upgamma =5$, the predictions where ${p}_{t}>0.4$ can hardly contribute to the loss. In contrast, when $\upgamma =1$, the predictions where ${p}_{t}>0.6$ still contribute to the loss. Meanwhile $\mathrm{\alpha }$ can also be used to re-weight the binary classes (has crack and has no crack) (Fig. 8 b-d). When $\mathrm{\alpha }$ is used for one class, the other class is re-weighted by $\left(1-\mathrm{\alpha }\right)$. Choosing a small $\mathrm{\alpha }$ for a class will obviously decrease the contribution of the whole class to the loss. For example, if $\mathrm{\alpha }=0.1$ is chosen for the “has crack” class and $\upgamma =5$, the predictions can be hardly improved when it is greater than $0.3$. Particularly, when $\upgamma = 0$ and $\mathrm{\alpha }= 0.5$, the FL is equivalent to the CE loss.

The dropout rate is another hyperparameter that influences the model performance. The value 0.75 is identified to yield the best performance among {0.25, 0.5, 0.75, 0.9}. This means the wave field information from only about 20 receiver locations are used during training. The reported results for hyperparameters $\upgamma$ and $\mathrm{\alpha }$ are based on this optimal dropout rate. A comparison of model performance with respect to all tested dropout rates can be found in Supplementary.

The hyperparameters $\upgamma$ and $\mathrm{\alpha }$ control the model's learning strength on “no crack” class and “has crack” class. As $\mathrm{\alpha }$ increases, the model is driven to focus on damaged cells, because the false predictions of damaged cells contribute more to the overall loss. As $\upgamma$ increases, the model is trained to focus on "hard" cells, where the model can't make predictions with high confidence. Because a higher $\upgamma$ value forces the model to pay more attention to the "hard" cells. The evaluations of models that are trained with different $\mathrm{\alpha }$ are given in Table 2. When assigning larger weights (larger $\mathrm{\alpha }$) to the “has crack” class for CE loss, the trained model tends to have lower precision and higher recall. This can be interpreted as the model's tendency to give more “has crack” predictions. On the contrary, using smaller $\mathrm{\alpha }$ for the “has crack” class results in higher precision but lower recall. This means the models tend to give less “has crack” predictions. When $\mathrm{\alpha }= 0.75$, the CE loss gives the model of the highest accuracy. The model is characterized as having balanced precision and recall, and also having the highest IoU and DSC metric values. The results using focal loss are shown in Table 3. By adding the penalty term, with carefully chosen $\upgamma$, the trained models have balanced the precision and recall, and thus result in an increase in IoU and DSC metrics. The accuracy is also improved compared to the models trained with CE loss. Specifically, the optimal model trained with CE loss ($\mathrm{\alpha }=0.75$) are improved slightly in comparing with the model trained with FL ($\mathrm{\alpha }=0.75$ $\upgamma =0.4$). The highest accuracy is achieved by the model trained with $\mathrm{\alpha }=0.9$ $\upgamma =0.1$ , however, with moderate IoU and DSC metric values.

**Table 2:** The evaluation results (including IoU, DSC, and accuracy) of models trained by varying $\alpha$ for CE loss ($\gamma$= 0, dropout rate 0.75).

${\upalpha }$	Prec	Recall	IoU	DSC	Accu
0.1	**0.932**	0.734	0.6969	0.821	0.753
0.2	0.911	0.777	0.722	0.839	0.772
0.2555	0.930	0.748	0.708	0.829	0.747
0.3	0.900	0.788	0.725	0.841	0.769
0.35	0.906	0.781	0.723	0.839	0.769
0.5	0.881	0.802	0.723	0.840	0.778
0.75	0.842	0.850	**0.733**	**0.846**	**0.813**
0.9	0.786	0.866	0.700	0.824	0.788
0.95	0.738	**0.870**	0.665	0.799	0.788

**Table 3:** The evaluation results (including IoU, DSC, and accuracy) of models trained by varying $\gamma$ for FL loss (optimal $\alpha$, dropout rate 0.75).

${\upgamma }$	${\upalpha }$	Prec	Recall	IoU	DSC	Accu
0	0.75	0.842	0.850	0.733	0.846	0.813
0.1	0.9	0.775	0.880	0.702	0.825	**0.822**
0.2	0.95	0.727	**0.895**	0.670	0.802	0.794
0.4	0.75	0.857	0.841	**0.738**	**0.849**	0.809
1	0.35	**0.882**	0.799	0.722	0.838	0.800
2	0.9	0.788	0.860	0.698	0.822	0.810
4	0.75	0.827	0.843	0.716	0.835	0.806

##### The selected thresholds

The accuracy is calculated dependently with two threshold settings: the threshold for crack existence in a pixel, and the threshold for correct prediction of a sample. The first threshold defines a probability value, above which a pixel is considered to contain a crack. In this work, it is referred to as binarizing threshold (${T}_{bin}$). The second threshold is to set “tolerance” ( ${T}_{tol}$) to count “accurate” predictions. The “tolerance” allows a prediction to be “accurate” when the predicted “has crack” pixels cover a certain area of the crack, i.e., its IoU score is greater than the threshold. The very strict criteria require that the predicted “has crack” pixels cover the true crack-existing area, i.e., the ${M}_{\text{IoU}}=1$, to be a “correct prediction”.

**Figure 9:**
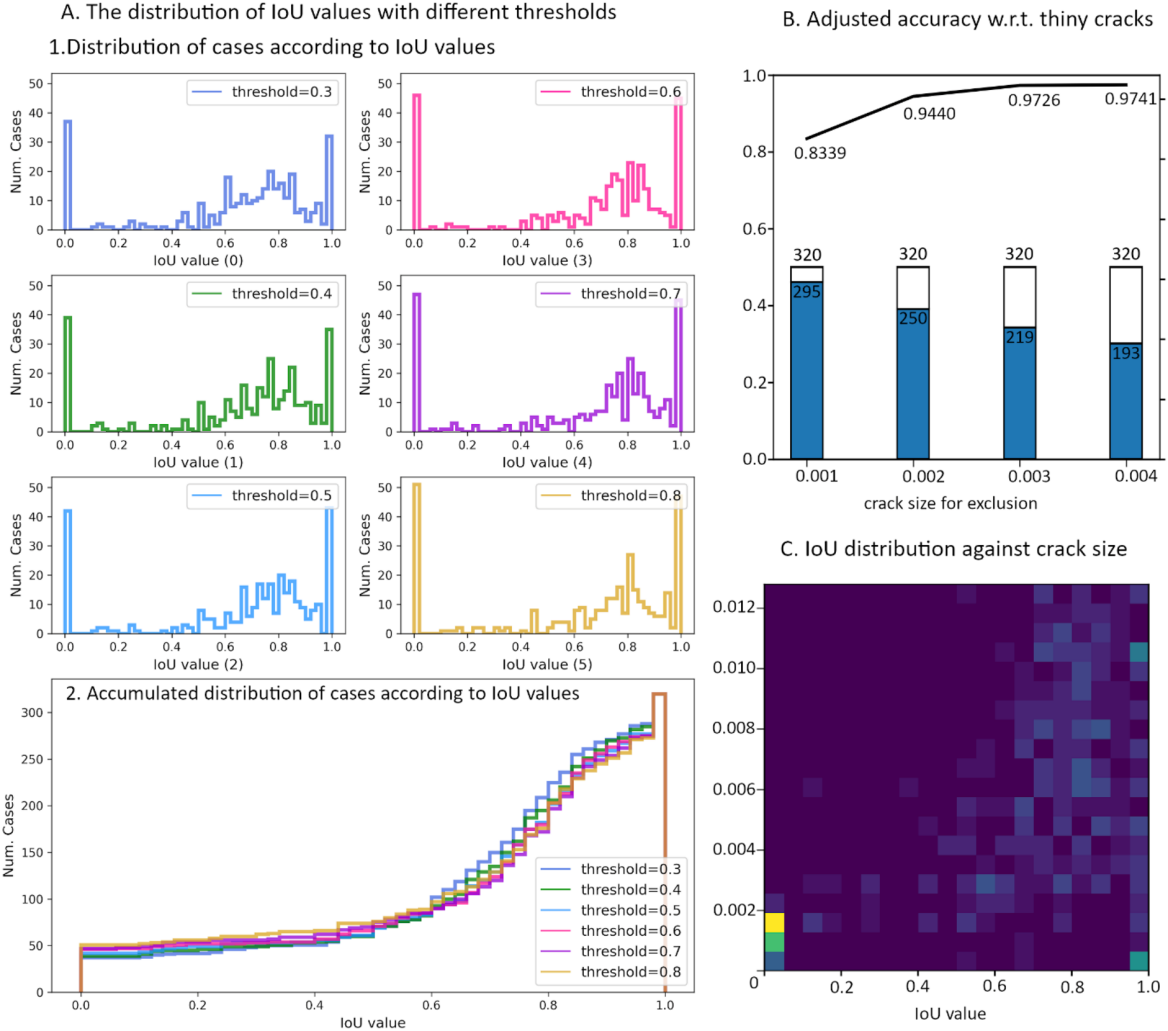
The relationship between crack size and evaluation metrics. The IoU values of 320 test cases are calculated by the model trained with $\gamma = 0.4$ and $\alpha = 0.75$. (**A**) 1. the 6 histograms of the IoU values that are calculated with 6 different binarization threshold ($T_{bin}$ = 0.3, 0.4, 0.5, 0.6, 0.7, 0.8); 2. the accumulated histograms of the IoU values that are calculated with 6 different thresholds. (**B**) The adjusted accuracy for test data after excluding tiny crack cases. The line plot presents the accuracy that is re-calculated when excluding cases with crack size less than 0.001, 0.002, 0.003, and 0.004; the bar chat shows the number of samples after excluding the samples with tiny cracks against the total number of samples in test dataset. (**C**) IoU values distribution w.r.t crack size.

**Figure 10:**
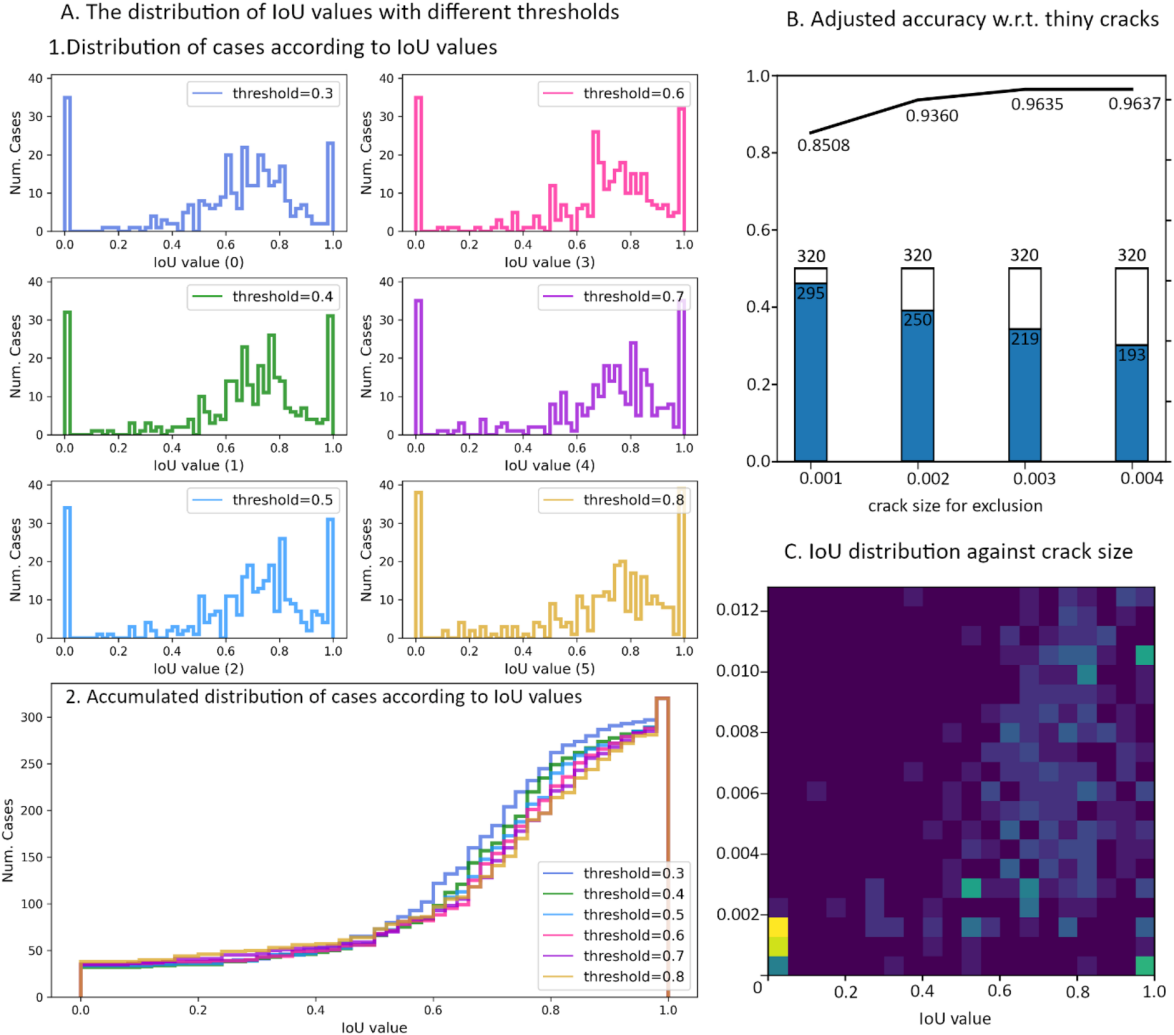
The relationship between crack size and evaluation metrics. The IoU values of 320 test cases are calculated by the model trained with $\gamma = 0.1$ and $\alpha = 0.9$. (**A**) 1. the 6 histograms of the IoU values that are calculated with 6 different binarization threshold ($T_{bin}$ = 0.3, 0.4, 0.5, 0.6, 0.7, 0.8); 2. the accumulated histograms of the IoU values that are calculated with 6 different thresholds. (**B**) The adjusted accuracy for test data after excluding tiny crack cases. The line plot presents the accuracy that is re-calculated when excluding cases with crack size less than 0.001, 0.002, 0.003, and 0.004; the bar chat shows the number of samples after excluding the samples with tiny cracks against the total number of samples in test dataset. (**C**) IoU values distribution w.r.t crack size.

The FL function pushes the predicted probabilities of “has crack” and “no crack” towards opposite extremes, because a sample with IoU value that is close to 0.5 will have a large penalty during training. This fact is also illustrated in Fig. 9 A and Fig. 10 A, which are resulted from the recommended model trained with $\mathrm{\alpha }=0.75$ $\upgamma =0.4$ and $\mathrm{\alpha }=0.9$ $\upgamma =0.1$. The sub-figures of Fig. 9 A.1 and Fig. 10 A.1 suggest that most damaged cells are correctly predicted with a probability above 0.5, while still-minor “hard cases” get a border prediction around 0.5, with about 45 cases that both models can not properly handle. In Fig. 9 A.2 and Fig. 10 A.2, the accumulated histograms show a clearer comparison on the quality of predictions for different ${T}_{bin}$ values. They show that different ${T}_{bin}$ produce similar accumulative histogram curves. This suggests that most "no crack" cells and many "has crack" cells are predicted with very high confidences. We can choose ${T}_{bin}=0.5$ as it also fits the configuration of FL loss. The curves begin to rise when IoU value reaches 0.5. This suggest us to chose ${T}_{tol}=0.5$ for evaluation, so that the number of cases with IoU values between 0 to 0.5 are relatively small while accumulate quickly when ${M}_{\text{IoU}}\ge 0.5$.

##### Discussion on model performance

The results in Figs. 9 A and 10 A indicate that both models are not good at detecting a minor set of damaged cases in test data. The minor set of hard cases can be characterized by crack size (percentage of “has crack” pixels in the 100 × 100 labeling image). In training data and test data, the samples with small cracks ($\le 0.004$) consist of a larger portion in test data then in training data (see Fig. S3). Figure 9 C and Fig. 10 C clearly show that the IoU values can be very low for samples with tiny cracks, while the IoU values for samples with larger crack are mostly above 0.5. The accuracy, adjusted by excluding samples with small crack size from test data, shown in Fig. 9 B and Fig. 10 B suggests that the proposed model is particularly good at identifying larger cracks ($> 0.004$). It indicates that the most low-quality predictions are made for samples with tiny cracks, while cases of larger crack sizes generally have better predictions. This means the developed model can easily distinguish between damaged cases and non-damage cases for large cracks but is not good at detecting tiny cracks. If we only count the cases with crack size greater than 0.002, the accuracy leaps by around 0.1. If we only count the cases with crack size greater than 0.004, the accuracy of both models can reach 0.95.

## Conclusion

The paper presents a new approach to detect damages by wave pattern recognition models. The major development is a learning CNN to detect on-hand the visible wave pattern of the damaged zone within a solid structure. To generate the cracked structure, a new dynamic Lattice Element method was used. The major advantage of this method is the application to heterogeneous structures under mechanical, hydraulically, thermal field influence and local chemical changes to describe the evolution of damages in solid structures. The use of new generation deep CNNs to analyse the time dependency within the changed wave pattern is promising. With the described method, a stable detection of 90 percent of the generated large cracks was possible. The next steps will be the reduction of the used number of receivers and increasing the model's ability of tiny crack detection.

## Supplementary Information

Supplementary Information.

## References

[1] Kaewunruen, S. & Remennikov, A., *Non-destructive Testing (NDT): A Tool for Dynamic Health Monitoring of Railway Track Structures*, presented at Materials Australia, North (2006).

[2] Farhangdoust S, Mehrabi A (2019). Health monitoring of closure joints in accelerated bridge construction: A review of non-destructive testing application. J. Adv. Concr. Technol..

[3] Azimi M, Eslamlou AD, Pekcan G (2020). Data-driven structural health monitoring and damage detection through deep learning: State-of-the-art review. Sensors.

[4] Kong X, Cai CS, Hu J (2017). The state-of-the-art on framework of vibration-based structural damage identification for decision making. Appl. Sci..

[5] Avci O (2021). A review of vibration-based damage detection in civil structures: From traditional methods to machine learning and deep learning applications. Mech. Syst. Signal Process..

[6] LeCun Y, Bengio Y, Hinton G (2015). Deep learning. Nature.

[7] Girshick, R., *Fast r-cnn**, *Presented at Proceedings of the IEEE international conference on computer vision (2015).

[8] Kim, Y., *Convolutional Neural Networks for Sentence Classification*, Presented at Proceedings of the 2014 Conference on Empirical Methods in Natural Language Processing (EMNLP) (2014). 10.18653/v1/d16-1076 PMC5300751 28191551

[9] van den Oord, A. *et al.*, *WaveNet: A Generative Model for Raw Audio*. Preprint at arXiv:1609.03499 (2016).

[10] Cha Y-J, Choi W, Büyüköztürk O (2017). Deep learning-based crack damage detection using convolutional neural networks. Comput. Aided Civ. Infrastruct. Eng..

[11] Feng D, Feng MQ (2018). Computer vision for SHM of civil infrastructure: From dynamic response measurement to damage detection—a review. Eng. Struct..

[12] Sha W, Edwards KL (2007). The use of artificial neural networks in materials science based research. Mater. Des..

[13] Guo T, Wu L, Wang C, Xu Z (2020). Damage detection in a novel deep-learning framework: a robust method for feature extraction. Struct. Health Monit..

[14] Khan A, Ko D-K, Lim SC, Kim HS (2019). Structural vibration-based classification and prediction of delamination in smart composite laminates using deep learning neural network. Compos. B Eng..

[15] Su C (2019). Improved damage localization and quantification of CFRP using lamb waves and convolution neural network. IEEE Sens. J..

[16] Gulgec NS, Takáč M, Pakzad SN (2019). Convolutional neural network approach for robust structural damage detection and localization. J. Comput. Civ. Eng..

[17] Sajedi SO, Liang X (2020). Vibration-based semantic damage segmentation for large-scale structural health monitoring. Comput. Aided Civ. Infrastruct. Eng..

[18] Abdeljaber O, Avci O, Kiranyaz S, Gabbouj M, Inman DJ (2017). Real-time vibration-based structural damage detection using one-dimensional convolutional neural networks. J. Sound Vib..

[19] Rautela, M. & Gopalakrishnan, S., *Deep Learning Frameworks for Wave Propagation-Based Damage Detection in 1D-Waveguides*, Presented at Proceedings of the 11th International Symposium NDT in Aerospace (2019).

[20] Rautela M, Gopalakrishnan S (2020). Ultrasonic guided wave based structural damage detection and localization using model assisted convolutional and recurrent neural networks. Expert Syst. Appl..

[21] Abdeljaber O (2018). 1-D CNNs for structural damage detection: Verification on a structural health monitoring benchmark data. Neurocomputing.

[22] Lin Y-Z, Nie Z-H, Ma H-W (2017). Structural Damage detection with automatic feature-extraction through deep learning. Comput. Aided Civ. Infrastruct. Eng..

[23] Rai A, Mitra M (2021). Lamb wave based damage detection in metallic plates using multi-headed 1-dimensional convolutional neural network. Smart Mater. Struct..

[24] Nunes, L. A., Amaral, R. P. F., de Souza Barbosa, F. & Cury, A. A., A hybrid learning strategy for structural damage detection. *Struct. Health Monit.* (2020).

[25] Wong, J. K. W., Soga, K., Xu, X. & Delenne, J.-Y., In *Modelling fracturing process of geomaterial Using Lattice Element Method*. 1700 (CRC Press, 2014)

[26] Rizvi, Z. H., Nikolić, M. & Wuttke, F., Lattice element method for simulations of failure in bio-cemented sands. *Granular Matter* **21** (2019).

[27] Sattari AS, Rizvi ZH, Motra HB, Wuttke F (2017). Meso-scale modeling of heat transport in a heterogeneous cemented geomaterial by lattice element method. Granular Matter.

[28] Rizvi, Z. H., Wuttke, F. & Sattari, A. S., *Dynamic Analysis by Lattice Element Method Simulation*, presented at Proceedings of China-Europe Conference on Geotechnical Engineering, Cham (2018).

[29] Rizvi, Z. H. *et al.*, *Dynamic Lattice Element Modelling of Cemented Geomaterials*, presented at Advances in Computer Methods and Geomechanics, Singapore (2020).

[30] Moukarzel C, Herrmann HJ (1992). A vectorizable random lattice. J. Stat. Phys..

[31] Wuttke F, Markwardt K, Schanz T (2012). Dispersion analysis in geotechnical laboratory tests: Time-frequency and time-scale signal transforms. Geotech. Test. J..

[32] Wuttke F, Asslan M, Schanz T (2012). Time-lapse monitoring of fabric changes in granular materials by coda wave interferometry. Geotech. Test. J..

[33] Minaee, S. *et al.*, Image segmentation using deep learning: A survey. *IEEE Trans. Pattern Anal. Mach. Intell.* 1–1 (2021). 10.1109/TPAMI.2021.3059968 33596172

[34] LeCun, Y., Bengio, Y. & others, Convolutional networks for images, speech, and time series. *The handbook of brain theory and neural networks* **3361**, 1995 (1995).

[35] Rumelhart DE, Hinton GE, Williams RJ (1986). Learning representations by back-propagating errors. Nature.

[36] Springenberg, J., Dosovitskiy, A., Brox, T. & Riedmiller, M., *Striving for Simplicity: The All Convolutional Net*, presented at ICLR (workshop track) (2015).

[37] Ioffe, S. & Szegedy, C., *Batch Normalization: Accelerating Deep Network Training by Reducing Internal Covariate Shift*, presented at International Conference on Machine Learning (2015).

[38] He, K., Zhang, X., Ren, S. & Sun, J., *Delving deep into rectifiers: Surpassing human-level performance on imagenet classification*, presented at Proceedings of the IEEE international conference on computer vision (2015).

[39] Srivastava N, Hinton G, Krizhevsky A, Sutskever I, Salakhutdinov R (2014). Dropout: A simple way to prevent neural networks from overfitting. J. Mach. Learn. Res..

[40] Noh, H., Hong, S. & Han, B., *Learning Deconvolution Network for Semantic Segmentation*, presented at Proceedings of the IEEE International Conference on Computer Vision (ICCV) (2015).

[41] Radford, A., Metz, L. & Chintala, S., *Unsupervised Representation Learning with Deep Convolutional Generative Adversarial Networks*, presented at 4th International Conference on Learning Representations, ICLR 2016, San Juan, Puerto Rico, May 2–4, 2016, Conference Track Proceedings (2016).

[42] Lin, T.-Y., Goyal, P., Girshick, R., He, K. & Dollar, P., *Focal Loss for Dense Object Detection*, presented at The IEEE International Conference on Computer Vision (ICCV) (2017).

[43] Ruder, S., An overview of gradient descent optimization algorithms. Preprint at arXiv:1609.04747 (2016).

[44] Kingma, D. P. & Ba, J., *Adam: A Method for Stochastic Optimization*, presented at 3rd International Conference on Learning Representations, ICLR 2015, San Diego, CA, USA, May 7–9, 2015, Conference Track Proceedings (2015).

