# Supplementary material for: Wave based damage detection in solid structures using spatially asymmetric encoder–decoder network
Source: Sci Rep. 2021 Oct 25;11:20968. doi: 10.1038/s41598-021-00326-2 (PMC8547223; doi:10.1038/s41598-021-00326-2)
Supplement: Supplementary file 1 — Supplementary Information. [file 41598_2021_326_MOESM1_ESM.pdf]

Wave based damage detection in solid structures using spatially asymmetric encoder-decoder network

Frank Wuttke[1,2,*], **Hao Lyu**[1,2,*], **Amir S. Sattari**[1], **and Zarghaam H. Rizvi**[1]

[1]Geomechanics and Geotechnics Group, Kiel University, Kiel, 24118, Germany
[2]Competence Centre for Geoenergy, Kiel University, Kiel, 24118, Germany
[*]frank.wuttke@ifg.uni-kiel.de
[*]hao.lyu@ifg.uni-kiel.de

Supplementary Information

Example of Simulated Displacement Wave Field

The Figures in section gives another example of simulated wave field. For the conditions in Fig.S1 - excitation point in upper middle boundary- the the wave field is plotted in Fig.S2.

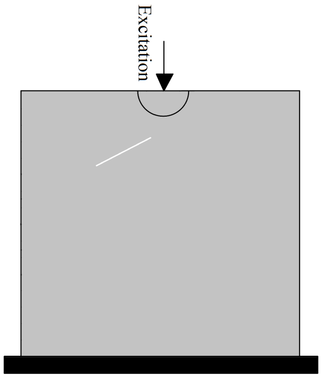

Figure S1. Boundary conditions: vertical excitation with generated crack.

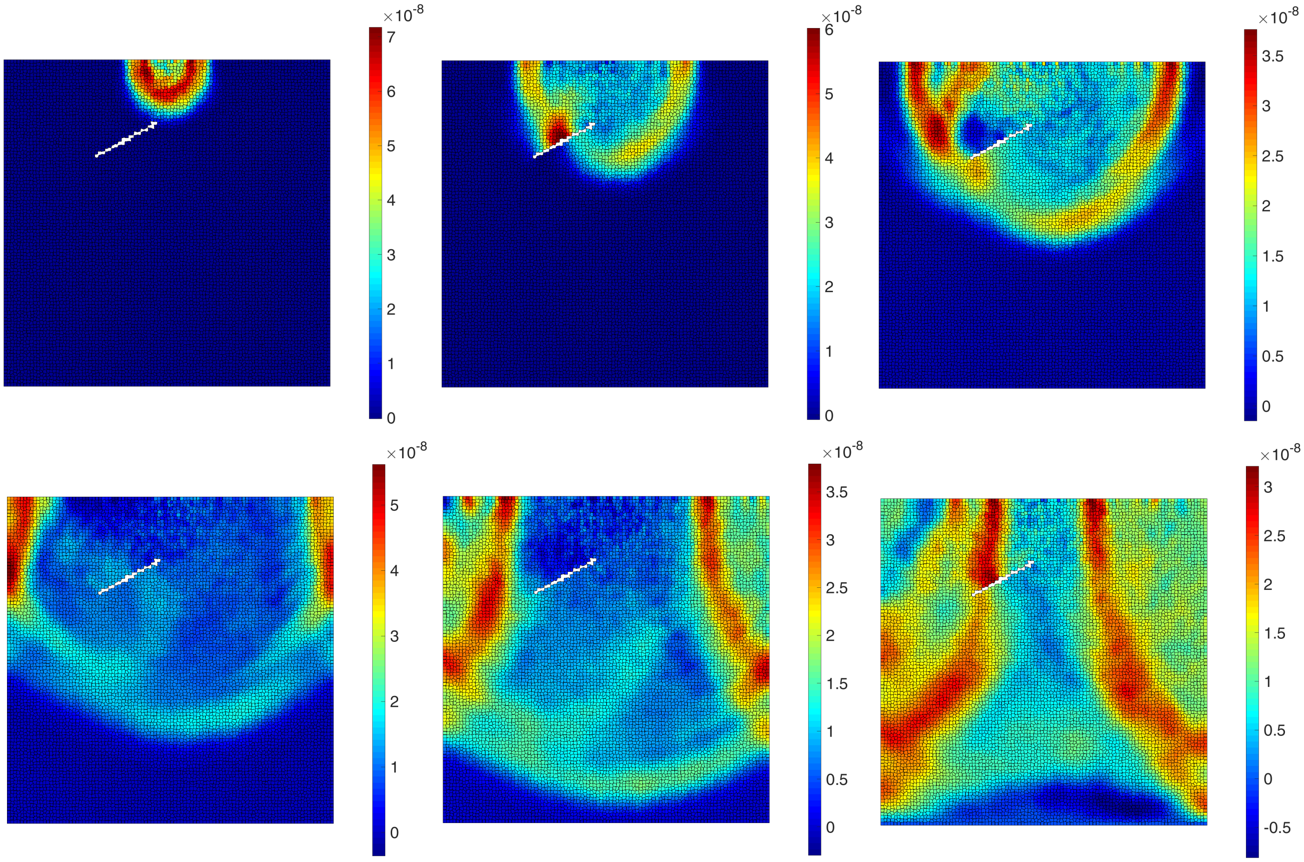

Figure S2. The 6 frames (100 time steps interval, from left to right) of a displacement (u_x) wave propagation inside the defined plate in Fig.S1

Damage Detection Dataset

Fig. S3 shows the number of cases with different crack sizes in training datas and test data. The number of cases with larger size cracks are slighly smaller than cases of smaller crack size. In test dataset the distribution of crack sizes is more balanced. The statistic information on crack size is given in Tab. S1. In Fig. S4, the 5 different case types (see Section "The Damage Detection Dataset") are marked with different coloured letters. This category can be used for further investigation on model performance w.r.t case types. Fig. S5 is the true occurrence of crack in full resolution (100 x 100) and Fig. S6 gives the true occurrence in a reduced resolution (16 x 16).

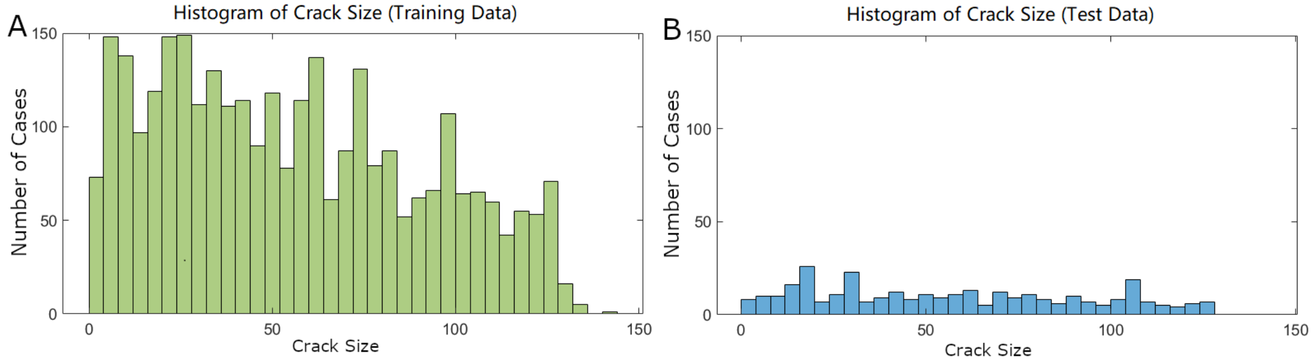

Figure S3. Histogram of crack size distribution in training data and testing data. A: crack size distribution in training data; B: crack size distribution in testing data.

dataset	mean	median	std.dev	min	max
train(16)	0.0515	0.0469	0.0266	0	0.1328
train(100)	0.0055	0.0051	0.0036	0	0.0143
test(16)	0.0525	0.0469	0.0268	0	0.1172
test(100)	0.0056	0.0052	0.0036	0	0.0128

Table S1. Crack size statistics. The crack size is measured as the ratio of the pixels that are labelled as damaged and total pixel number of the labelling image.

Example of Crack Detection Predictions

Figures in this section show true crack occurrence and predictions made by the best-performing model for 320 test cases in 16 columns and 20 rows. The models in this work are trained based on the labels of 16x16 resolution. The output in Fig. S7 - S10 are made by two models which are trained with focal loss of $\alpha = 0.75$ $\gamma = 0.4$ and $\alpha = 0.9$ $\gamma = 0.1$, the two recommended alpha and gamma values we found in our experiments. During training both models, the dropout rate is set to 0.75, and the learning rate is set to Adam's default value of 0.001. Fig. S7 and S9 show the predicted probability of crack existence for each pixel, a brighter pixel indicates a higher probability of crack inside. While in Fig. S8 and S10, binary predictions with the threshold that the pixel with probability greater than 0.5 is considered as having a crack inside.

	1	2	3	4	5	6	7	8	9	10	11	12	13	14	15	16
1	C	C	C	S	C	C	C	C	C	C	C	N	N	N	N	N
2	C	C	C	S	C	C	C	C	C	C	C	N	N	N	N	N
3	C	C	C	S	C	C	C	C	C	C	C	N	N	N	N	N
4	C	C	C	N	C	C	C	C	C	C	C	N	N	N	N	N
5	C	C	C	N	C	C	C	C	C	C	C	N	N	N	N	N
6	C	C	C	N	C	C	C	C	C	C	C	N	N	N	N	N
7	C	C	C	N	C	C	C	C	C	C	C	N	N	N	N	N
8	C	C	C	N	C	C	C	C	C	C	C	N	N	N	N	R
9	C	C	SC	N	C	C	C	C	C	C	N	N	N	N	N	R
10	C	C	SC	N	C	C	C	C	C	C	N	N	N	N	N	R
11	C	C	SC	N	C	C	C	C	C	C	N	N	N	N	N	R
12	C	C	SC	N	C	C	C	C	C	C	N	N	N	N	N	R
13	C	C	SC	N	C	C	C	C	C	C	N	N	N	N	N	R
14	C	C	SC	N	C	C	C	C	C	C	N	N	N	N	N	R
15	C	C	SC	N	C	C	C	C	C	C	N	N	N	N	N	R
16	C	C	S	N	C	C	C	C	C	C	N	N	N	N	N	N
17	C	C	S	C	C	C	C	C	C	C	N	N	N	N	N	N
18	C	C	S	C	C	C	C	C	C	C	N	N	N	N	N	N
19	C	C	S	C	C	C	C	C	C	C	N	N	N	N	N	N
20	C	C	S	C	C	C	C	C	C	C	N	N	N	N	N	N

Type-C C Type-N N Type-R R Type-S S Special Cases SC

Figure S4. The category for 320 test cases. The test cases are categorised into four types: 1). randomly generated samples with randomly generated cracks (*Type-N*), 2). randomly generated samples with no crack (*Type-R*), 3). randomly generated with similar cracks (*Type-S*), and 4). the same sample with different cracks (*Type-C*). They are marked by the colored marks. The special cases (marked as "SC" in yellow) are the 7 cases we intentionally generated with the same cracks in training data but from different samples.

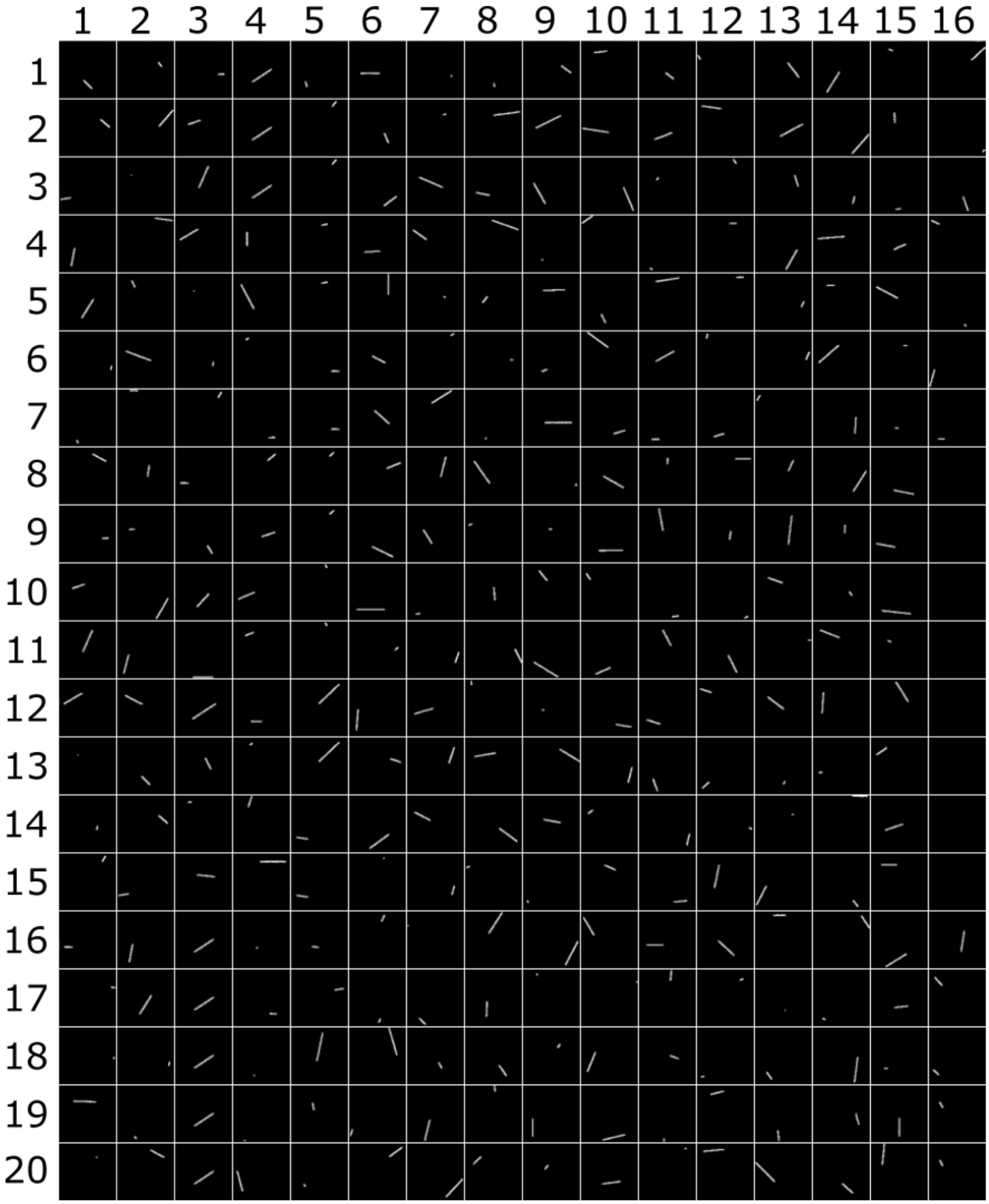

Figure S5. The true crack occurrence in reduced resolution (100 x 100) for 320 testing cases.

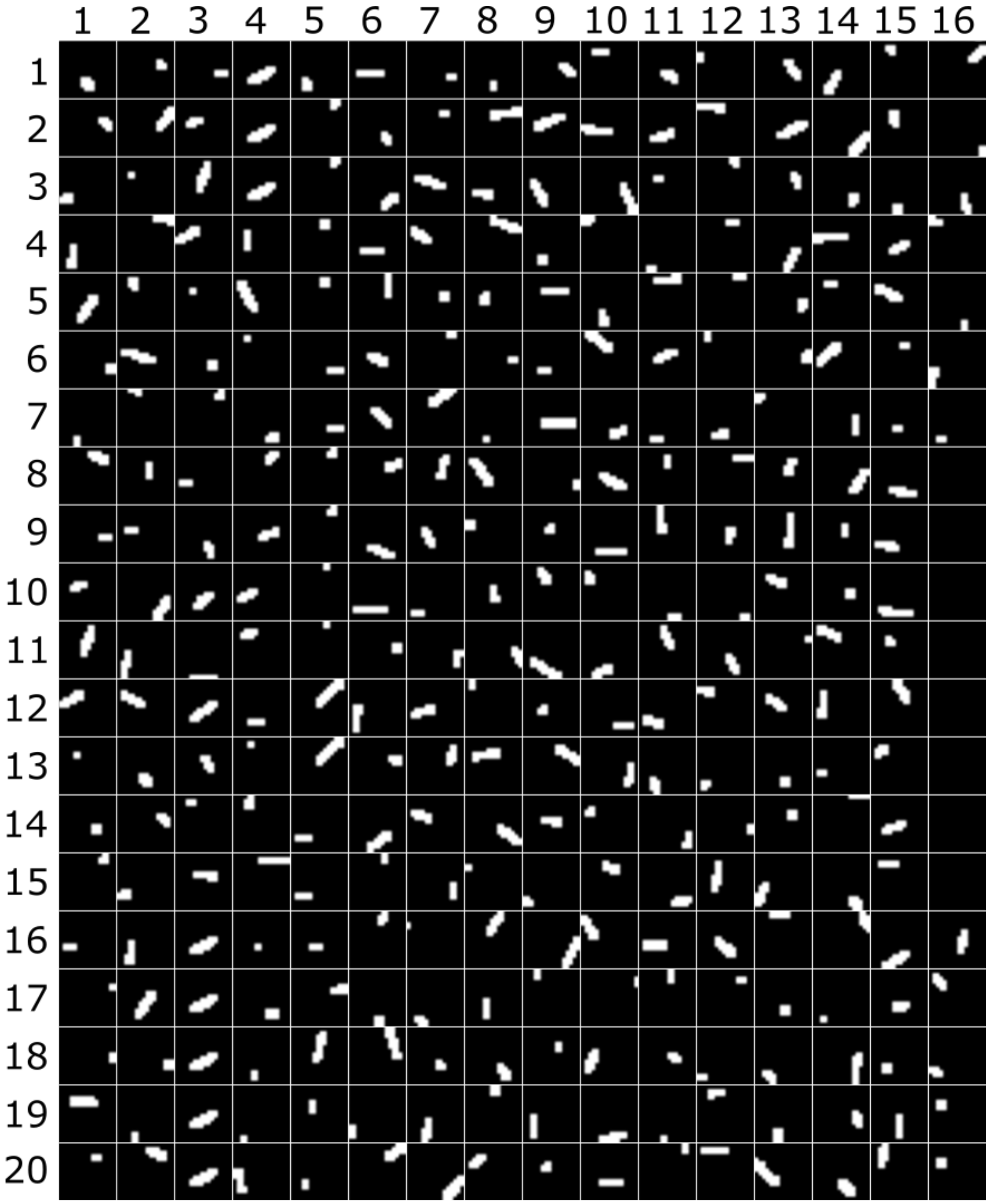

Figure S6. The true crack occurrence in reduced resolution (16 x 16) for 320 testing cases.

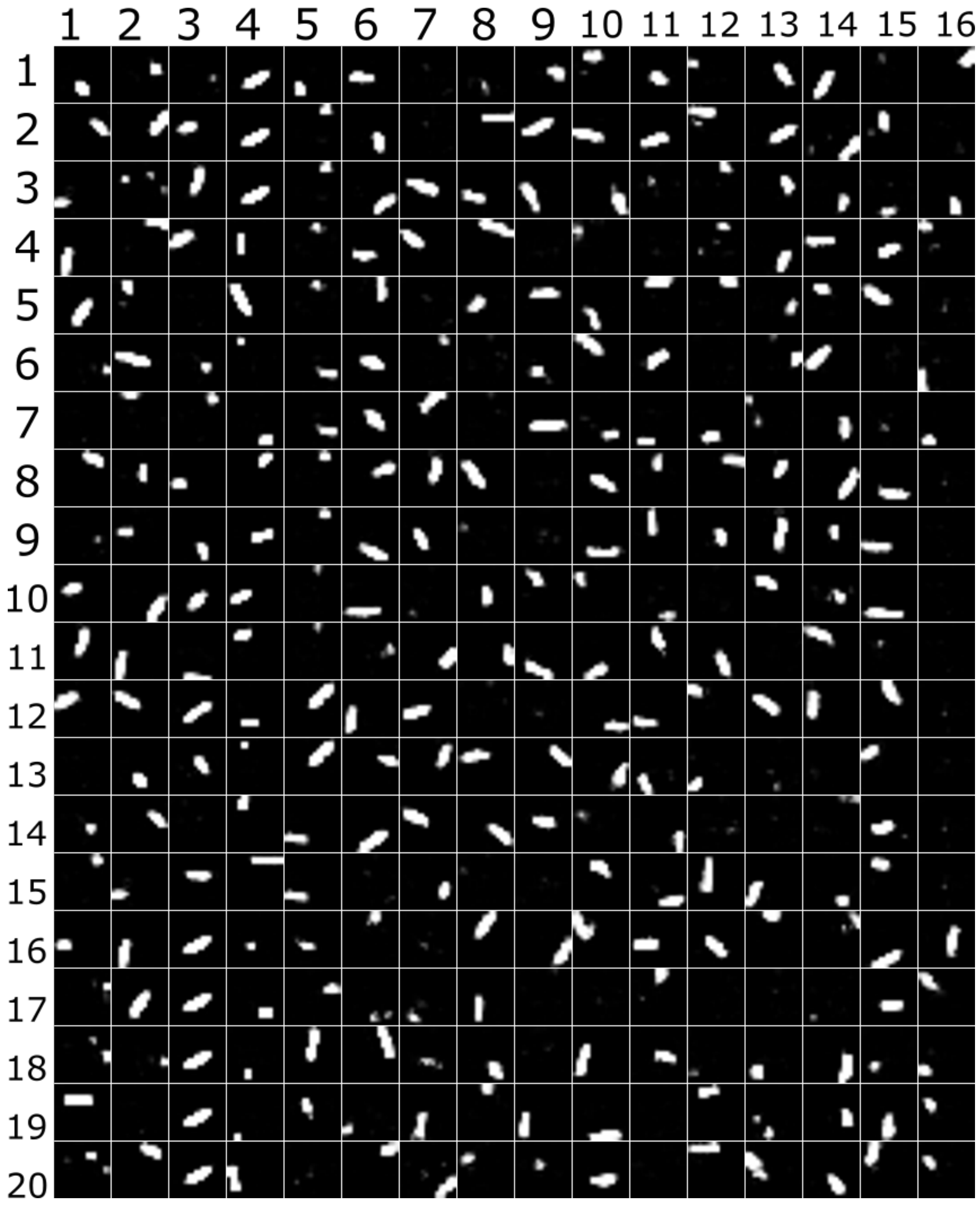

Figure S7. The predicted probability of crack existence in pixels for 320 test cases, the brighter a pixel's color is indicates the higher probability of crack existence inside the pixel. The model is trained with dropout rate 0.75, $\alpha = 0.75$ and $\gamma = 0.4$

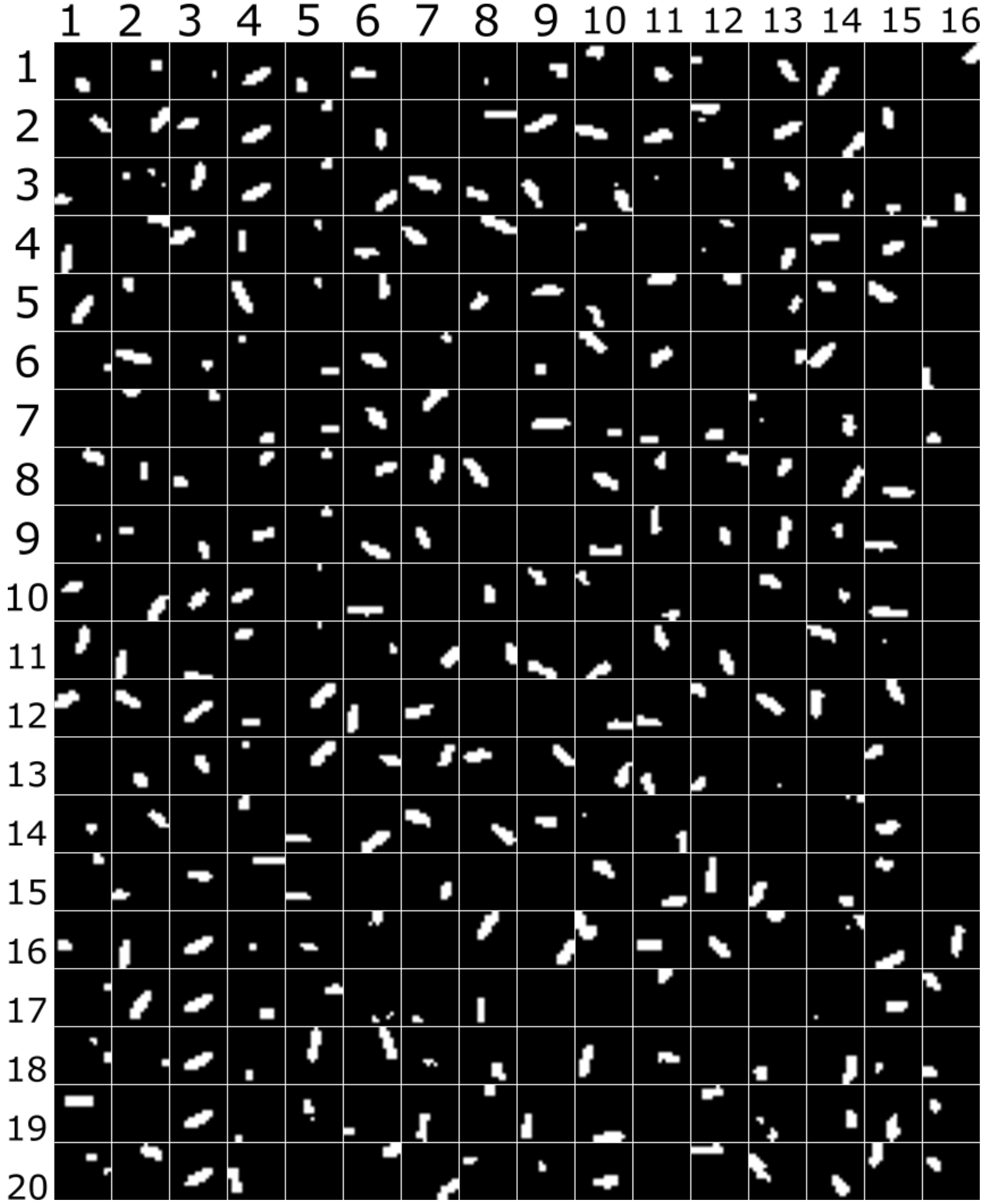

Figure S8. The binary predictions made by the model trained with dropout rate 0.75, $\alpha = 0.75$ and $\gamma = 0.4$, where the pixel with a probability greater than a certain threshold (0.5) is considered as having a crack inside.

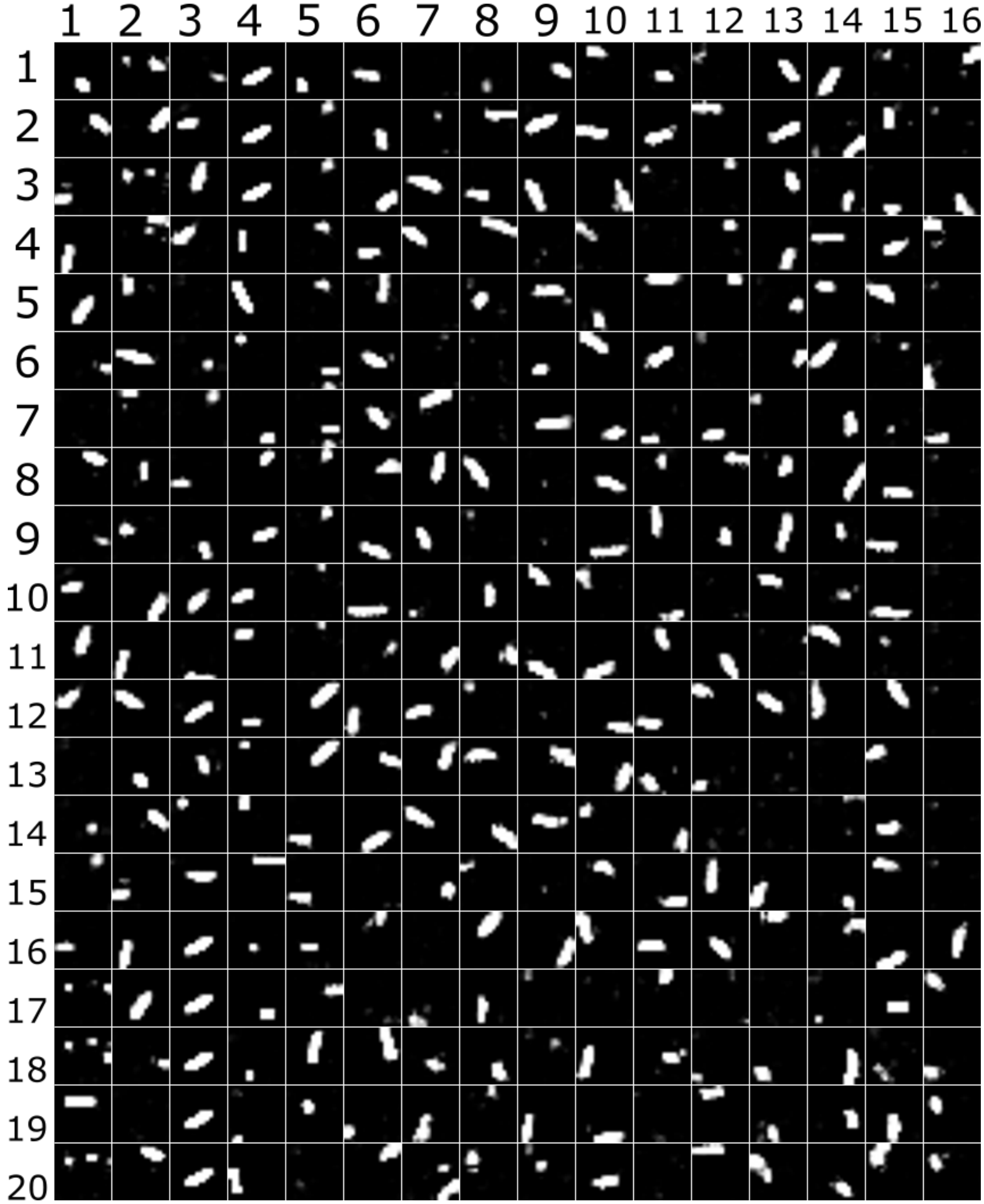

Figure S9. The predicted probability of crack existence in pixels for 320 testing cases, the brighter a pixel's color is indicates the higher probability of crack existence inside the pixel. The model is trained with dropout rate 0.75, $\alpha = 0.9$ and $\gamma = 0.1$

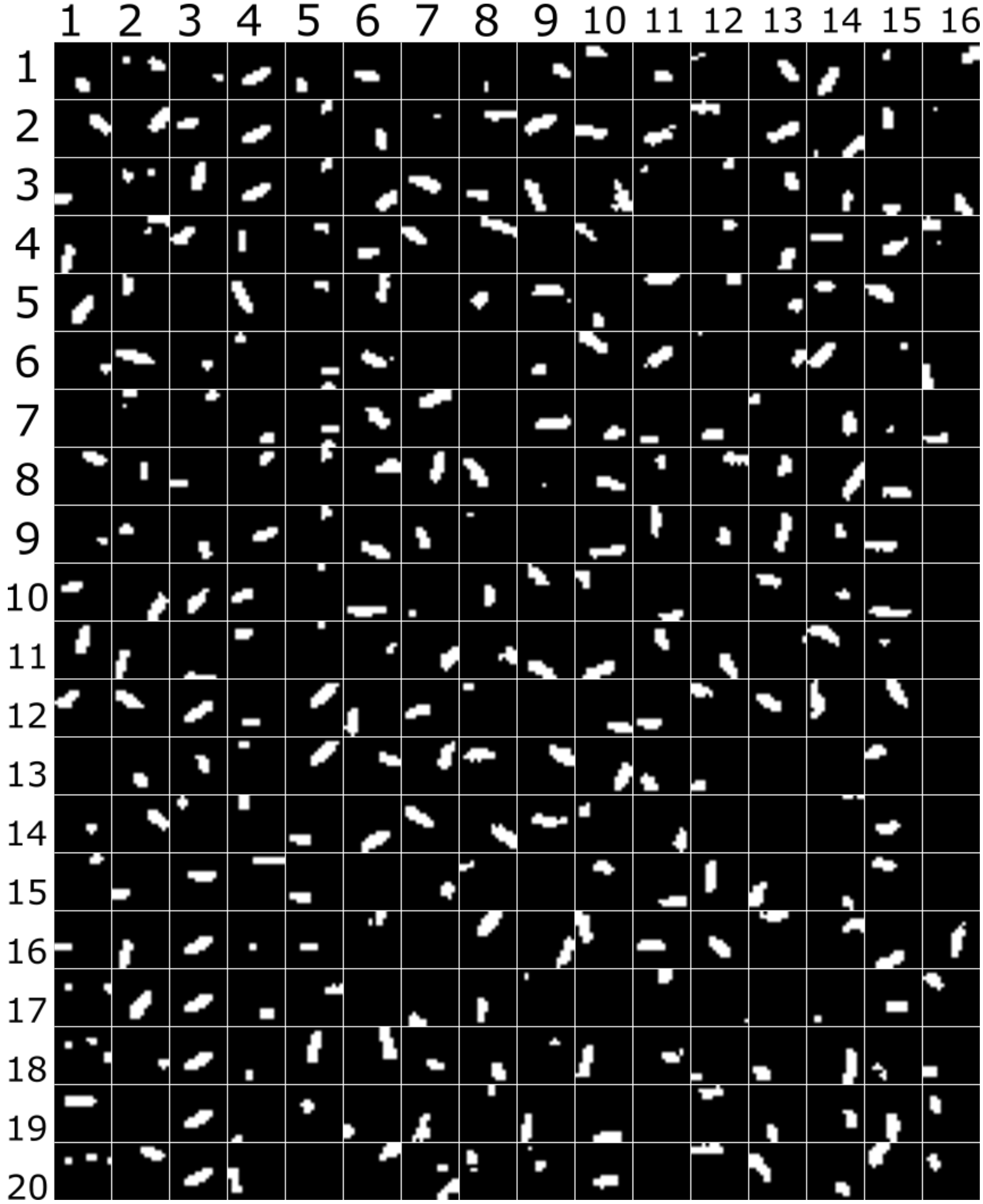

Figure S10. The binary predictions made by the model trained with dropout rate 0.75, $\alpha = 0.9$ and $\gamma = 0.1$, where the pixel with probability greater than a certain threshold (0.5) is considered as having a crack inside.

Training With/Without Dropout

Comparing the relatively small amount of training data (ca. 3,000 samples) with the number of parameters of the proposed neural network (more than 600,000). Preventing overfitting plays a key role in successful training. Overfitting is the phenomenon that after a certain training iteration, improving the model's fit to the training data also increases the generalization error. It can be characterized as a continuous decreasing in the training loss curve and a rising in the validation loss curve after that iteration. The commonly used strategies for handling overfitting include increasing the amount of data, data augmentation, early stop, and adding a dropout layer in the network structure. Data augmentation increases the amount of data by applying some transformations or slight modifications to existing data. It's commonly used for image data[1], however, it's difficult to find a physically sound transformation for the simulated data in this study. The early stop is a regularization technique of estimating the point where overfitting occurs[2]. Training procedures can be stopped around the point to avoid overfitting. Dropout is another widely used regularization technique in training neural network models[3,4]. The key idea of "Dropout" is to randomly drop nodes and their connections from the neural network during training to prevent complex co-adaptations on training data. The resulted model can be viewed as an average of a series of models generated by the random drop strategy. In this study, we choose to randomly "dropout" all features from the same receiver location. The resulted model can be seen as an average of a series of models that is trained with randomly selected receiver locations.

In the following part, we show a set of figures on the model performance and training/validation curves with respect to different learning rates of Adam optimizer without using Dropout, as well as the results from the models trained with Adam's default learning rate (0.001) with Dropout. Fig. S11 shows the 4 models of optimal α and γ values under the 4 different learning rates, each is trained 100 epochs. It suggests that the three major metrics, i.e., accuracy, IoU, and DSC, slightly decrease as the 4 selected learning rate values { 0.001, 0.00075, 0.0005, 0.0002 } decrease. The trend is obvious, especially for learning rate value 0.0002. However, when looking into Fig. S12, it clearly shows a sign of overfitting when the learning rate is among 0.001 (Fig. S12 A), 0.00075 (Fig. S12 B), and 0.0005 (Fig. S12 C). Besides, the curves have large drops and rises during training, indicating an unstable training. Choosing a learning rate value of 0.0002 (Fig. S12 D), leads to a slight decrease in accuracy, but also prevents severe overfitting. It seems strange when checking the metric curves in the 4 sub-figures (Fig. S12 A-D), that the accuracy, IoU, and DSC value remain more or less stable even after overfitting occurs. The rising validation loss curve be explained as the overfitted model has lower confidence in its prediction on the validation data, while the maitaining metric curves suggest that the predictions for most pixels don't excced the binary threshold, and thus remain correct.

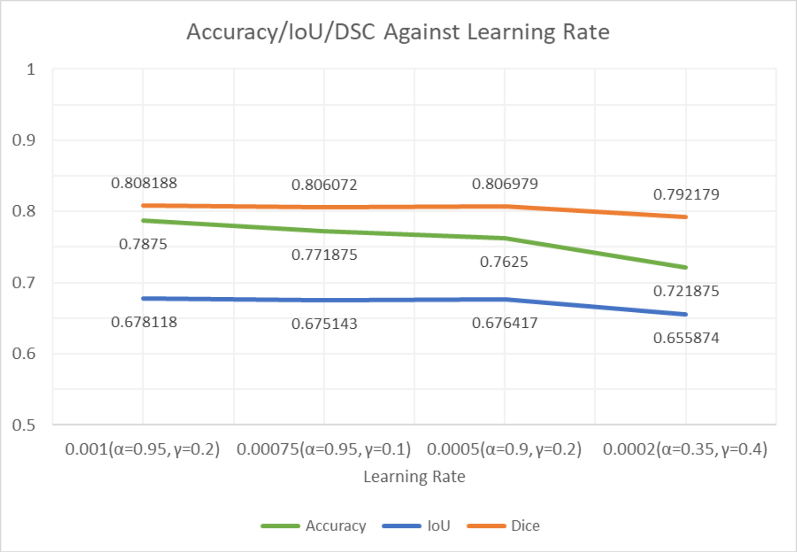

Figure S11. The highest IoU, DSC, and Accuracy values from the models trained with different learning rate, without using Dropout.

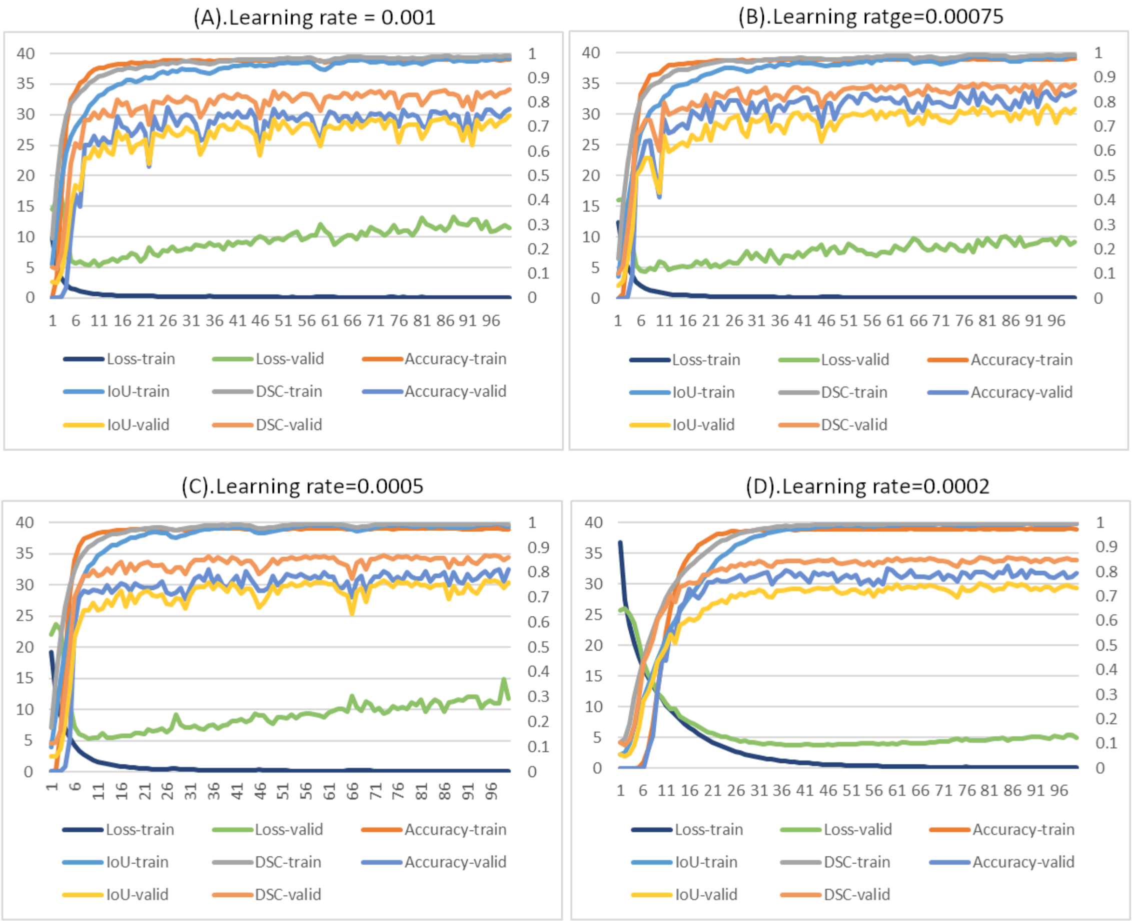

Figure S12. The traing/validation curves as well as metrics of different learning rate without using Dropout.

To identify a proper dropout rate, 4 values (0.25, 0.5, 0.75, 0.9) are tested. All the models have trained 150 epochs with Adam's default learning rate value 0.001. Dropout rate depicts the average percentage of the nodes to be dropped in one layer. The models are trained with the set of 4 dropout values under varying α and γ. The learning rate value remains unchanged as Adam's default. Optimal models trained with the 4 dropout rates are reported here. Fig. S13 illustrates that a proper dropout rate (≥ 0.5) increases the model performance. The metric curves peak at the dropout rate value of 0.75 and drops down slightly when the dropout rate is 0.9. Averagely, when the dropout rate is set to 0.75, about 20 receivers' data are finally used to make predictions, while about 8 receivers' data are finally used when the dropout rate is set to the value of 0.9. This suggests that when too many receivers' data is used at once, the training gets overfitting quickly, while using too few receivers during training also has a negative effect on the model's performance. Fig. S14 show the training/validation curves of the 4 models chosen for Fig. S13. Adopting the dropout technique in the model makes the training more robust and stable and prevents severe overfitting.

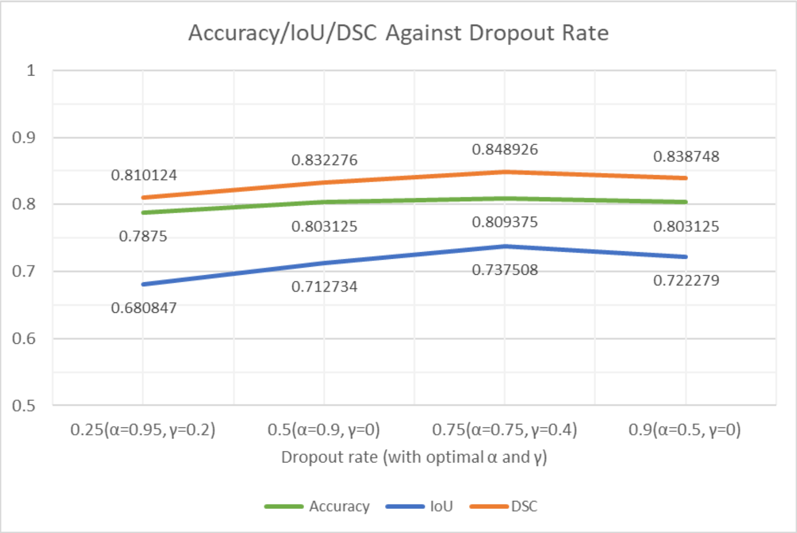

Figure S13. The highest IoU, DSC, and Accuracy values from the models trained with different dropout rate.

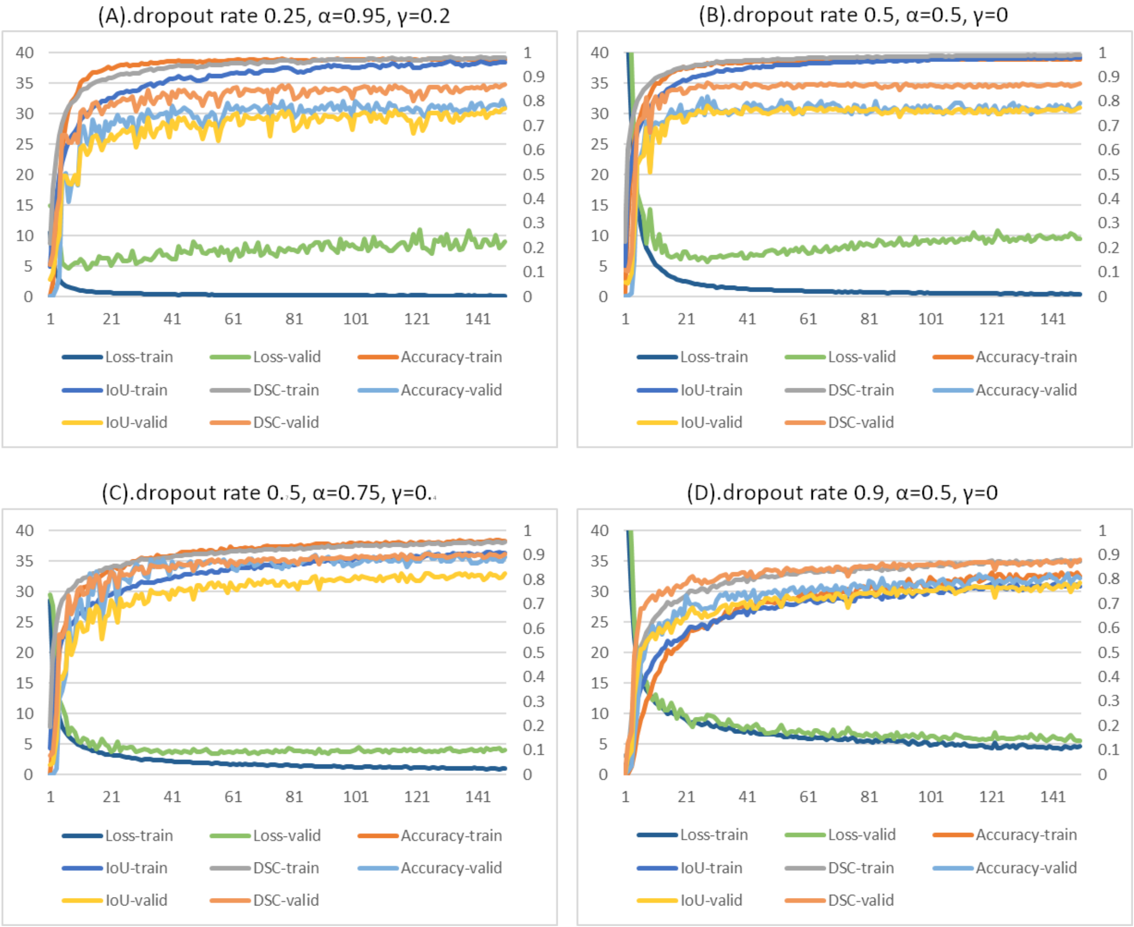

Figure S14. The highest IoU, DSC, and Accuracy values from the models trained with 4 different dropout rate (0.25, 0.5, 0.75, 0.9) and the same learning rate (0.001).

Training With Reduced Amount of Receivers

To study the effect of the number of receivers in building the crack detection model, 4 reduced receiver grids as shown in Fig. S15 are tested. They are characterized as 5x5 (25), 3x3 (9), 2x2 (4) and 1 in the figure from left to right. We tested two variations of the proposed model on the reduced receiver grids. The reduced receiver grides are still regular ones, however, both variations have the ability to handle irregular receiver displacement. We additionally check the performance of the two variations on the full receiver grid (9x9) as a reference.

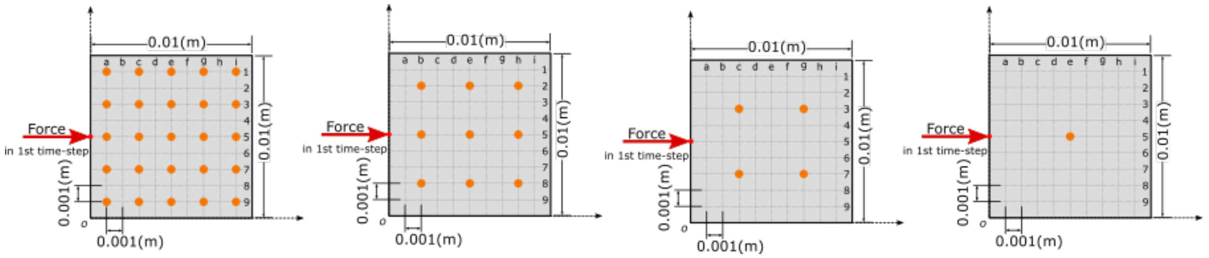

Figure S15. Configuration for the reduced receiver arrangement on the surface of a plate.From left to right show the 5x5, 3x3, 2x2, and 1 receiver grids on the sample surface.

The naive method is to adjust the nodes of the model's input layer following the number of used receivers. This won't change the WP layers as they perform 1D-convolution on each receiver location independently. The Fusion block, which brings the information from all receiver locations together, needs to be modified. With too few receivers, the two 2D-ConvLayers in the fusion block don't fit the spatial dimension of the input features anymore. Two Dense layers are thus used instead of the two 2D-ConvLayers. This is the only modification for the naive variation. The other parts of the network architecture, as well as kernel sizes, filter numbers, and strides, remain the same. The detailed implementation is illustrated in Fig. S16.

Finally, three sets of meaningful models are obtained with 4, 9, and 25 receivers. No meaningful result can be achieved within 150 training epochs using only 1 receiver. Dropout is not applicable for this model as no receiver location will be selected when the dropout rate is less than 1. Additionally, the network variation is also trained with full receivers (81) as a reference. The 4 models trained with 4, 9, 25, and 81 receivers employ the same α, γ, and learning rate (0.001). The dropout rate in all trainings is set to 0.75, which means for 4 receivers, an average of 1 receiver is used per sample during training. For 9 and 25 receivers, an average of 2 and 6 receivers are used per sample during training accordingly. Fig. S17 shows the accuracy, IoU, and DSC metrics with varying receiver numbers. As the number of receivers decreases, the model performance decreases as well. Fig. S18 shows the curves of the metrics in the training procedure for the reported 4 models. With the help of dropout, no severe overfitting happened. Using too few receivers also causes larger drops and rises in the curve ((Fig.S18 A). Using fewer receivers makes the training convergence slower, and it seems the model could be further improved for more training epochs ($\geq$ 150) (Fig.S18 A, B).

Depending on the results reported here, we can conclude that reducing the receivers to a certain amount still results in sound models while using more numbers of receivers with dropout strategy can help to increase the model's performance.

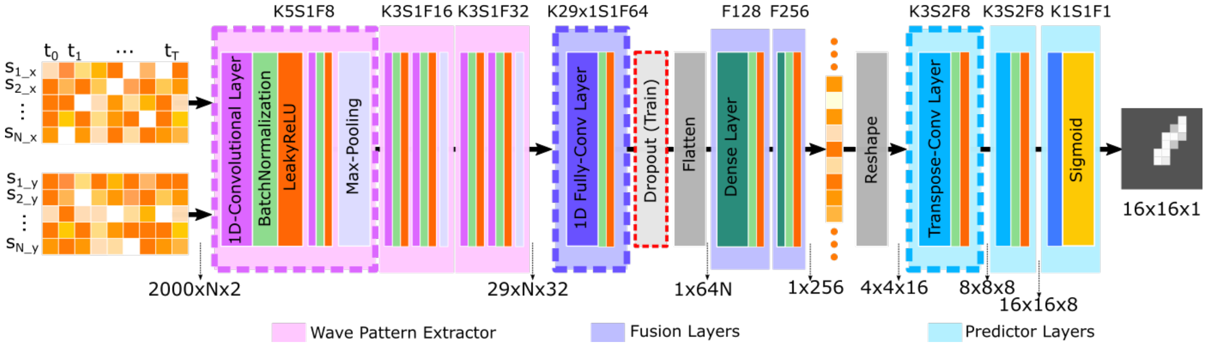

Figure S16. The first variation of the damage detection model for reduced amount of receivers. The shape of input data and layer output is placed at the bottom; layer configurations are placed on top for each layer. N represents the actual number of receivers in use.

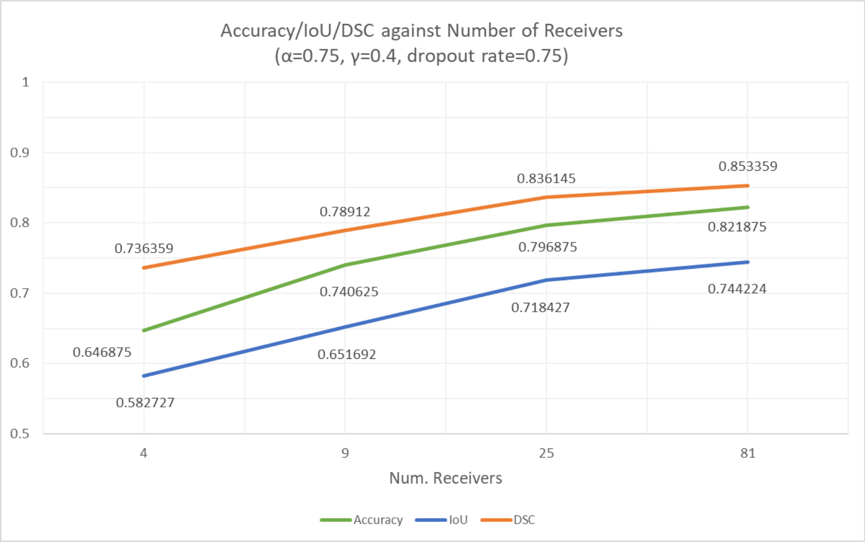

Figure S17. The Accuracy, IoU and DSC of models trained with reduced number of receivers (4, 9, 25), and full number of receivers(81). The presented 4 models are trained with the same hyperparameters, $\alpha = 0.75$, $\gamma = 0.4$, learning rate 0.001, dropout rate 0.75.

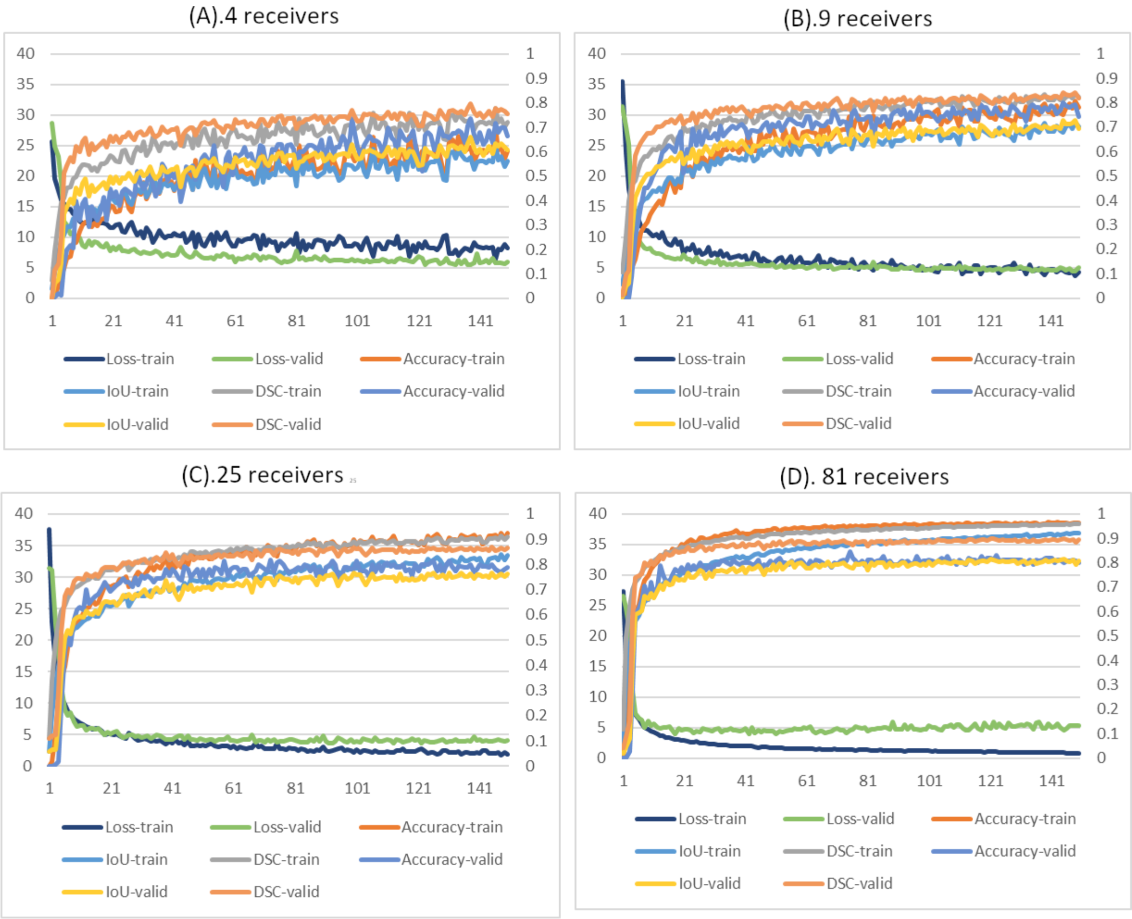

Figure S18. The training/validation curves for the 4 model represented in Fig. S17.

The second variation is designed to use a mask-dropout layer. The original dropout layer randomly drops some parts of its input features in training and returns the identity otherwise. The idea of mask-dropout layer is to use a predefined mask to drop features from unwanted receiver locations. In this case, the input shape maintains the same, while the data from the dropped receiver locations can be set to 0. The resulted variation is only to substitute the normal dropout layer with the masked version. The idea of mask-dropout is shown in Fig. S19, where $\mathbf{F}$ represents the input features, $\mathbf{X}'$ and $\mathbf{M}$ are randomly generated and predefined masks. The elementwise multiplication of masks and input features sets the value of dropped features to 0 (In practice, the kept values are scaled up accordingly). S20 show the value of the metrics from the mask-dropout variation trained under different dropout rates. Using a larger dropout rate forces the model to learn as much as possible from individual sensors. When using a value of 0.9, averagely about 8 sensors are used in training, so the resulted model performs well using 9 sensors configuration or more sensors. On the other side, the use of dropout value 0.5, which takes averagely about 40 sensors for training, resulted in the models that only perform well using the full sensor configuration (81 sensors) and not so good performance using less than 40 (25, 9, and 4) sensors.

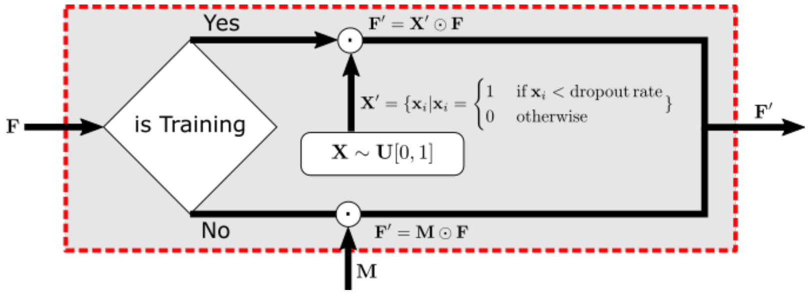

Figure S19. The mask-dropout layer of the second variation for reduced amount of receivers.

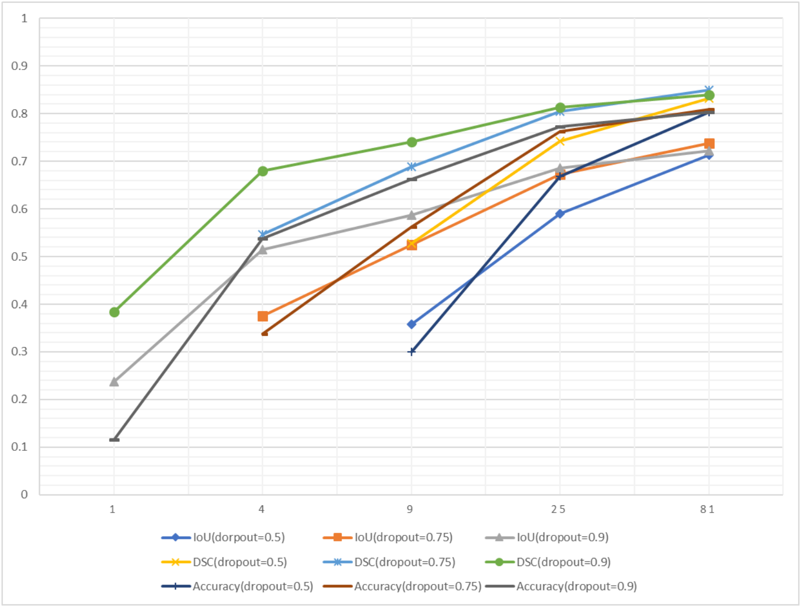

Figure S20. The accuracy, IoU, and DSC values against 5 configurations of the masked receiver locations. The values are taken from the models of optial α and γ.

To summarize, both variations yield acceptable models when wave field information from only several receivers' locations are available. Both variations have their own pros and cons, the naive variation yields a slightly better model than the one using mask-dropout layer. However, it can't maintain the geometric locations of the receivers, and thus needs to be modified according to the configuration of the recievers' number and location. The variation, which employs the mask-dropout layer, maintains receivers' geometric locations and thus is able to handle varying receiver configurations. To achieve a good performance, both variations need to be trained with carefully selected hyperparameters, α, γ, and dropout rate. The selection of dropout rate has a very large influence on the variatons' applicability to different receivers' configurations.

References

1. Shorten, C. & Khoshgoftaar, T. M. A survey on image data augmentation for deep learning. *J. Big Data* **6**, 1–48, DOI: https://doi.org/10.1186/s40537-019-0197-0 (2019).
2. Prechelt, L. *Early Stopping — But When?*, 53–67 (Springer Berlin Heidelberg, Berlin, Heidelberg, 2012).
3. Hinton, G. E., Srivastava, N., Krizhevsky, A., Sutskever, I. & Salakhutdinov, R. R. Improving neural networks by preventing co-adaptation of feature detectors. *arXiv preprint arXiv:1207.0580* (2012).
4. Srivastava, N., Hinton, G., Krizhevsky, A., Sutskever, I. & Salakhutdinov, R. Dropout: A simple way to prevent neural networks from overfitting. *J. Mach. Learn. Res.* **15**, 1929–1958 (2014).
